# Radiomic features and tumor immune microenvironment associated with anaplastic lymphoma kinase-rearranged lung adenocarcinoma and their prognostic value

**DOI:** 10.3389/fgene.2025.1581937

**Published:** 2025-05-01

**Authors:** Ying Han, Wenya Feng, Huaxin Li, Hua Wang, Zhaoxiang Ye

**Affiliations:** ^1^ Departments of Biotherapy, Tianjin Medical University Cancer Institute and Hospital, National Clinical Research Center for Cancer, Tianjin’s Clinical Research Center for Cancer, State Key Laboratory of Druggability Evaluation and Systematic Translational Medicine, Key Laboratory of Cancer Immunology and Biotherapy, Tianjin, China; ^2^ Departments of Radiology, Tianjin Medical University Cancer Institute and Hospital, National Clinical Research Center for Cancer, Tianjin’s Clinical Research Center for Cancer, State Key Laboratory of Druggability Evaluation and Systematic Translational Medicine, Key Laboratory of Cancer Prevention and Therapy, Tianjin, China; ^3^ Department of Diagnostic Radiology, Tianjin Cancer Hospital Airport Hospital, Tianjin, China

**Keywords:** radiomics, tumor microenvironment, lung adenocarcinoma, anaplastic lymphoma kinase, computed tomography

## Abstract

**Purpose:**

To identify radiomic features from preoperative computed tomography (CT) images and characteristics of the tumor immune microenvironment (TIME) associated with anaplastic lymphoma kinase (*ALK*) rearrangement in patients with lung adenocarcinomas and their prognostic value in predicting recurrence or metastases after surgery.

**Materials and methods:**

This retrospective study included 66 *ALK*-positive and 66 *ALK*-negative patients who underwent surgical resected lung adenocarcinoma. The number of CD8^+^ T cells and Human leukocyte antigen class I (HLA-I)/programmed death ligand 1 (PD-L1) expression were determined using immunohistochemistry. Radiomic features were extracted from the preoperative CT images. Combined radiomic, clinicopathological, and clinicopathological-radiomic models were built to predict *ALK* rearrangements. The models’ prediction performance was analyzed using receiver operating characteristic (ROC) curves with five-fold cross-validation. Prediction models for determining disease-free survival (DFS) of *ALK*-rearranged patients were developed, and the C-index after internal cross-validation was calculated to evaluate the performance of the models.

**Results:**

HLA-I and PD-L1 expression were negatively associated with *ALK* rearrangement (both P < 0.001). The ROC curve indicated areas under the curve of 0.763, 0.817, and 0.878 for the radiomics, clinicopathology, and combined models in predicting *ALK* rearrangement, respectively. The combined model showed significant improvement compared to the clinicopathological (P = 0.02) and radiomics (P < 0.001) models alone. The validation C-indices were 0.752, 0.712, and 0.808 for the radiomic, clinicopathological, and combined models in predicting the DFS of *ALK*-rearranged patients, respectively. The combined model showed a significant improvement (P < 0.001) compared to the clinicopathological model alone.

**Conclusion:**

This study demonstrated the potential role of radiomics and TIME characteristics in identifying *ALK* rearrangements in lung adenocarcinomas and the prognostic value of radiomics in predicting DFS in patients with *ALK* rearrangements.

## 1 Introduction

Anaplastic lymphoma kinase (*ALK*) rearrangements are driver mutations that occur in approximately 3%–7% of non-small cell lung cancer (NSCLC) cases, primarily in the lung adenocarcinoma subtype (Qin and Gadgeel, 2017; Smolarz et al., 2025). Compared with other tumor genotypes, *ALK*-rearranged tumors exhibit more invasive histomorphological features and aggressive behaviors (Kim et al., 2013). *ALK* rearrangements are an important target for NSCLC treatment, and patients harboring *ALK* rearrangements receive significant clinical benefits from *ALK* tyrosine kinase inhibitors (TKIs) (Shaw et al., 2013).

Although targeted therapies are effective in patients with *ALK* rearrangements, drug resistance and tumor recurrence inevitably occur (Rothenstein and Chooback, 2018). Immune checkpoint inhibitors (ICIs) that block the programmed death-1 (PD-1)–programmed death ligand 1 (PD-L1) axis have demonstrated remarkable therapeutic effects against NSCLC (Borghaei et al., 2015; Brahmer et al., 2015; Reck et al., 2016). However, studies have shown that patients harboring *ALK* rearrangements do not benefit from ICIs (Gainor et al., 2016; Mazieres et al., 2019; Jahanzeb et al., 2021). This may be associated with the unique tumor immune microenvironment (TIME).

Human leukocyte antigen class I (HLA-I) plays a pivotal role in tumor neoantigen presentation and CD8^+^ T cell activation. Some studies have suggested that activation of oncogenes inhibits HLA-I expression, thus promoting immune escape and thereby contributing to the poor efficacy of immunotherapy (Brea et al., 2016; Watanabe et al., 2019).

Although previous studies have reported increased tumor PD-L1 expression to be an unfavorable prognostic factor for NSCLC, the characteristics of the TIME and their prognostic values in *ALK*-rearranged NSCLC remain unclear (Zhang et al., 2017; Zhang et al., 2022; Tian et al., 2023; Zhou et al., 2023).

Through the extraction of high-throughput quantitative characteristics from medical images acquired during clinical practice, radiomics can offer insights into unique phenotypes resulting from the underlying biological processes of a tumor (Tomaszewski and Gillies, 2021). These radiomic features can noninvasively provide comprehensive information about the microenvironmental heterogeneity of tumors, and radiomics-based biomarkers have been widely used to predict clinical outcomes and potential genomic patterns (Chen et al., 2017). However, few studies have been conducted on the radiomic features of NSCLC with *ALK* rearrangement, and none have examined the association between radiomic features and disease-free survival (DFS) after surgery in this specific population (Ninatti et al., 2020; Ma and Li, 2021; Chen et al., 2024).

Patients with *ALK*-rearranged lung adenocarcinoma exhibit low response rates to ICIs, potentially due to distinct TIME characteristics. Radiomics, by noninvasively quantifying tumor heterogeneity, may improve early detection and risk stratification, thus complementing conventional pathology. Thus, in this study, we aimed to (I) identify the preoperative computed tomography (CT) radiomic features and TIME characteristics associated with *ALK* rearrangement in lung adenocarcinomas, (II) determine their potential value in predicting recurrence or metastasis after surgery in patients with *ALK* rearrangement, and (III) examine whether a combination of radiomic features and TIME characteristics could improve the performance of the predictive model. This multimodal analysis may aid in the early identification of *ALK* rearrangement and risk stratification for *ALK*-positive patients and provide a rationale and guidance for tailored therapy in the early stages.

## 2 Materials and methods

### 2.1 Study population

The institutional review board of Tianjin Medical University Cancer Institute and Hospital approved this retrospective study (Ethical approval No. EK20240091). Patients provided written informed consent prior to undergoing tests related to pathology, immune microenvironment, and *ALK* rearrangement status. This analysis included patients who underwent surgical resection for lung cancer in Tianjin Medical University Cancer Institute and Hospital between July 2016 and December 2019. Patients were consecutively included based on the following criteria: (I) Histologically confirmed lung adenocarcinoma with *ALK* rearrangement detected by Ventana D5F3 immunohistochemistry (IHC) in radical resection specimen, and (II) preoperative thin-slice CT images available on the Picture Archiving and Communication System conducted <1 month before surgery. Patients who had received chemotherapy or radiotherapy before surgery or those with other primary malignant tumors were excluded. Finally, 66 *ALK*-positive patients were included and 66 *ALK*-negative patients were randomly selected as controls (Supplementary Figure S1). Clinicopathological features, including sex, age, smoking history, and pathological TNM stage, were extracted from patients’ medical records. The tumors were histologically staged according to the eighth edition of the TNM classification system of the International Union Against Cancer and American Joint Committee on Cancer (Detterbeck et al., 2017). Patients were followed-up after surgery until December 2023. DFS was defined as the time from surgery to tumor recurrence or metastasis, which was confirmed by an investigator blinded to the predictor variables. Follow-up data were obtained from the medical records and telephone interviews. Among the *ALK*-positive patients, four received TKI treatment before tumor recurrence. Three patients without follow-up data were excluded from the prognostic analyses.

### 2.2 IHC

IHC staining was performed on paraffin-embedded sections of surgical samples using methods described previously (Mu et al., 2022). Primary antibodies included those against HLA-I (1:1,000; clone no. EMR8-5; cat. no. ab70328; Abcam, Cambridge, United Kingdom), PD-L1 (1:10,000; clone 2B11D11; cat. no. 66 248-1-Ig; Proteintech, Rosemont, IL, United States), and CD8 (1:10,000; clone no. 1G2B10; cat. no. 66 868-1-Ig; Proteintech).

Two pathologists who were blinded to the *ALK* status or clinical outcome independently evaluated all IHC images, and the final result was obtained by averaging the values from both pathologists. In this study, the H-score was used to evaluate the expression of HLA-I or PD-L1 on the cell membranes of tumor cells (Mu et al., 2022; Greeshma et al., 2023). The H-score was calculated as follows (Equation 1):

$$\begin{array}{c} H-\text{score}=0\times \%\text{ of nonstained tumor epithelial cells}\\ +1\times \%\text{ of weakly stained tumor epithelial cells}\\ +2\times \%\text{ of moderately stained tumor epithelial cells}\\ +3\times \%\text{ of strongly stained tumor epithelial cells} \end{array}$$ (1)

The number of CD8^+^ tumor-infiltrating lymphocytes (TILs), defined as CD8-positive cells regardless of the staining intensity, was recorded, and the density of TILs was determined by dividing the number of TILs by the total area of the observed fields (mm^2^) (Haratani et al., 2017). Five fields per section were randomly selected at ×200 magnification, avoiding necrotic and non-tumor regions, to calculate H-scores and CD8^+^ T cell density. The average values were then calculated.

### 2.3 CT imaging and radiomics feature extraction

Preoperative chest CT was performed using one of three multidetector CT systems: SOMOATOM Definition AS+ (Siemens Healthineers, Erlangen, Germany), LightSpeed 16 (GE Healthcare, Chicago, IL, United States), or Discovery CT750 HD (GE Healthcare). The scanning parameters were as follows: tube voltage, 120 kVp; tube current, 150–200 mA with automatic exposure control; reconstruction thickness and interval, 1.5 or 1.25 mm; mediastinal window reconstruction kernel, B30f/Standard; and lung window reconstruction kernel, B70f/lung.

Tumor segmentation was performed by a radiologist with 5 years of experience in thoracic CT diagnosis using a semiautomatic method, and reviewed by another radiologist with 16 years of experience. In addition to lung cancer diagnosis, the radiologists were unaware of clinical data and pathological information. 3D Slicer V5.1.0 (Fedorov et al., 2012) was used to segment tumors on unenhanced images using the B70f/lung reconstruction kernel. The B70f/lung kernel was selected because of its high resolution in capturing tumor edges and internal structures in the lung window, which is ideal for radiomic analysis. Three-dimensional (3D) radiomic features were extracted.

Finally, 851 features were extracted from the tumor CT images, as described in a previous study (Wang et al., 2022), including 14 shape features, 18 first-order features, 75 texture features [24 Gy-level co-occurrence matrix (GLCM), 14 Gy-level dependence matrix (GLDM), 16 Gy-level run-length matrix (GLRLM), 16 Gy-level size-zone matrix (GLSZM), and 5 neighboring gray-tone difference matrix (NGTDM)], and 744 wavelet-based features.

### 2.4 Feature selection and model development

#### 2.4.1 Models for predicting *ALK* rearrangement

Three models (radiomics, clinicopathological, and clinicopathological-radiomics combined) were developed separately to predict *ALK* rearrangement.

For radiomic model development, preliminary screening was performed using univariate logistic regression, and statistically significant features were further screened using the least absolute shrinkage and selection operator (LASSO) regression methods. Ten-fold cross-validation was applied to select the penalty parameter (λ) of LASSO via minimum criteria to retain features with nonzero coefficients. Finally, multivariate logistic regression was performed using a forward stepwise strategy to select the most informative variables in a single parsimonious model. The radiomics score (Rad-score) for each patient was calculated as a linear combination of the selected features weighted by their regression coefficients (beta values).

To build the clinicopathological model, univariate logistic regression analysis was performed, and statistically significant variables were included in a multivariate logistic regression model. A combined model was eventually developed by incorporating the independent predictive variables in the clinicopathological model and Rad-score into the multivariate logistic regression analysis.

The prediction performance of the models was analyzed using receiver operating characteristic (ROC) curves, and each model was cross-validated with five-fold cross-validation to ensure a robust area under the curve (AUC) estimate, given the limited sample size. Differences in the AUC between the models were compared using the DeLong test. Model stability was evaluated via the coefficient of variation (CV) of the AUCs derived from 500 bootstrap iterations (CV = standard deviation/mean).

#### 2.4.2 Models for predicting the DFS of patients with *ALK* rearrangement

Three models (radiomics, clinicopathological, and clinicopathological-radiomics combined) were developed separately to predict the DFS of patients with *ALK* rearrangements.

For the radiomics model, univariate Cox proportional hazards regression analysis was performed, and statistically significant features were subjected to LASSO Cox regression. Features with nonzero coefficients selected by 10-fold cross-validation were included in the backward stepwise Cox regression analysis to identify independent prognostic variables and to build the final model. A radiomics risk score (RAD-risk score) was calculated for each patient via a linear combination of selected features weighted by their regression coefficients (beta values). Patients with *ALK* rearrangements were divided into two risk groups based on the median RAD-risk score, which served as the cutoff point.

Clinicopathological variables that were significantly associated with DFS were identified using univariate and multivariate Cox regression analyses to construct a clinicopathological model. The combined model was finally developed by adding the RAD-risk score to the clinicopathological model in the multivariate analysis.

The Harrel concordance index (C-index) was used to assess the model performance. Owing to the limited sample size, five-fold cross-validation was used to ensure a robust C-index estimate. The 95% confidence intervals (CIs) for the C-index were calculated by bootstrap resampling (500 replicates). Differences in the C-index between models were assessed using a likelihood ratio test. Model stability was assessed through the CV of the C-indices from 500 bootstrap iterations.

### 2.5 Statistical analyses

Differences between *ALK*-positive and *ALK*-negative patients in terms of HLA-I/PD-L1 H-score, CD8^+^ T-cell density, and other clinicopathological features were evaluated using the Chi-squared test for categorical variables and the Mann–Whitney U test for continuous variables.

To further elucidate the biological relevance of the radiomic signature, the association of the calculated Rad-score and RAD-risk score with clinicopathological features was analyzed using the Spearman correlation test for continuous variables and the Mann–Whitney U test for categorical variables.

Statistical analyses were performed using R version 4.3.2 (The R Foundation for Statistical Computing), Python version 3.12 (Python Software Foundation, Wilmington, DE, United States), and SPSS version 27.0 (IBM Corp., Armonk, NY, United States). Differences were considered statistically significant at P < 0.05.

## 3 Results

### 3.1 Clinicopathological features stratified by *ALK* rearrangement status

The distribution of clinicopathological features according to *ALK* rearrangement status is presented in Table 1. The HLA-I and PD-L1 H-scores were significantly lower (both P values < 0.001) in patients with *ALK* rearrangements than in those without *ALK* rearrangements. There were no significant differences in sex, age, smoking history, T stage, N stage, pathological stage, or CD8^+^ T cell density between the two groups.

**TABLE 1:** Clinicopathological features stratified by *ALK* rearrangement statu**s**.

Clinicopathological feature	*ALK*+	*ALK*-	*p*-value
Sex			0.38
Male	35 (53.0)	30 (45.5)	
Female	31 (47.0)	36 (54.5)	
Age (years)	58 (14)	58 (13)	0.93
Smoking history			0.08
Never	44 (66.7)	34 (51.5)	
Ever	22 (33.3)	32 (48.5)	
T stage			>0.99
T1	48 (72.7)	48 (72.7)	
T2-4	18 (27.3)	18 (27.3)	
N stage			0.25
N0	44 (66.7)	50 (75.8)	
N1-2	22 (33.3)	16 (24.2)	
Pathological stage			0.46
Ⅰ	43 (65.2)	47 (71.2)	
Ⅱ-ⅢA	23 (34.8)	19 (28.8)	
HLA-I H-score	20 (64.50)	90 (167.75)	**<0.001**
PD-L1 H-score	23 (75)	88 (114.25)	**<0.001**
CD8^+^ T cell density	12 (10.25)	11 (9.25)	0.83

Data presented as N (%) or median (interquartile range).

Bolded values indicate a statistically significant result.

Continuous variables (age, HLA-I H-score, PD-L1 H-score, and CD8^+^ T cell density) were compared using the Mann-Whitney U test, whereas categorical variables (sex, smoking history, T stage, N stage, and pathological stage) were analyzed using the Chi-square test.

Abbreviations: *ALK*, anaplastic lymphoma kinase; HLA-I: human leukocyte antigen class I; PD-L1: programmed death ligand 1.

### 3.2 Models for predicting *ALK* rearrangement

A total of 122 radiomic features associated with *ALK* rearrangements were initially identified using univariate logistic regression (Supplementary Table S1) and screened using LASSO. The optimal λ was calculated to be 0.013, corresponding to 17 features with nonzero coefficients (Supplementary Figure S2; Supplementary Table S2). Forward stepwise regression analysis identified four robust radiomic features as independent predictors of *AL*K rearrangement (Table 2). A prediction model based on the four radiomic features was constructed, and the Rad-score for each patient was calculated. The Rad-score calculation formula is as follows (Equation 2, Supplementary Material 1):

$$\begin{array}{l} \text{Rad}-\text{score}=-0.015\;–\;0.777\times o\text{riginal}.\text{GLCM}.\text{Autocorrelation}\\ +\;0.648\times \text{Wavelet}\_\text{LLH}.\text{GLSZM}.\text{SmallAreaEmphasis}\\ +0.742\times \text{Wavelet}\_\text{HLL}.\text{GLCM}.\text{Correlation }\\ –\;1.185\times \text{Wavelet}\_\text{LLL}.\text{Firstorder}.\text{Skewness} \end{array}$$ (2)

**TABLE 2:** Multivariate logistic regression analyses of radiomic features to predict *ALK* rearrangement.

Radiomic feature	Beta value	Odds ratio (95% CI)	*p*-value
Original.GLCM.Autocorrelation	−0.777	0.46 (0.21–1.00)	0.050
Wavelet_LLH.GLSZM.SmallAreaEmphasis	0.648	1.91 (1.23–2.98)	0.004
Wavelet_HLL.GLCM.Correlation	0.742	2.10 (1.33–3.32)	0.001
Wavelet_LLL.Firstorder.Skewness	−1.185	0.31 (0.14–0.68)	0.004

Abbreviations: *ALK*, anaplastic lymphoma kinase; CI, confidence interval; GLCM, gray-level cooccurrence matrix; GLSZM, gray-level size zone matrix.

Among the clinicopathological features, univariate logistic regression analysis revealed that HLA-I and PD-L1 were significantly associated with *ALK* rearrangements (Supplementary Table S3). These two features were included in a multivariate logistic regression analysis, which indicated that the independent predictive features were HLA-I [odds ratio (OR) = 0.99; 95% CI: 0.98–0.99; P < 0.001] and PD-L1 (OR = 0.99; 95% CI: 0.98–0.99; P < 0.001), and these were incorporated into the establishment of a clinicopathological model. After combination with the Rad-score, the multivariate analysis showed that the significant factors in the combined model were Rad-score (OR = 2.88; 95% CI: 1.82–4.88; P < 0.001), HLA-I (OR = 0.99; 95% CI: 0.98–0.99; P < 0.001), and PD-L1 (OR = 0.99; 95% CI: 0.98–0.99; P < 0.001) (Supplementary Table S4).

**FIGURE 1:**
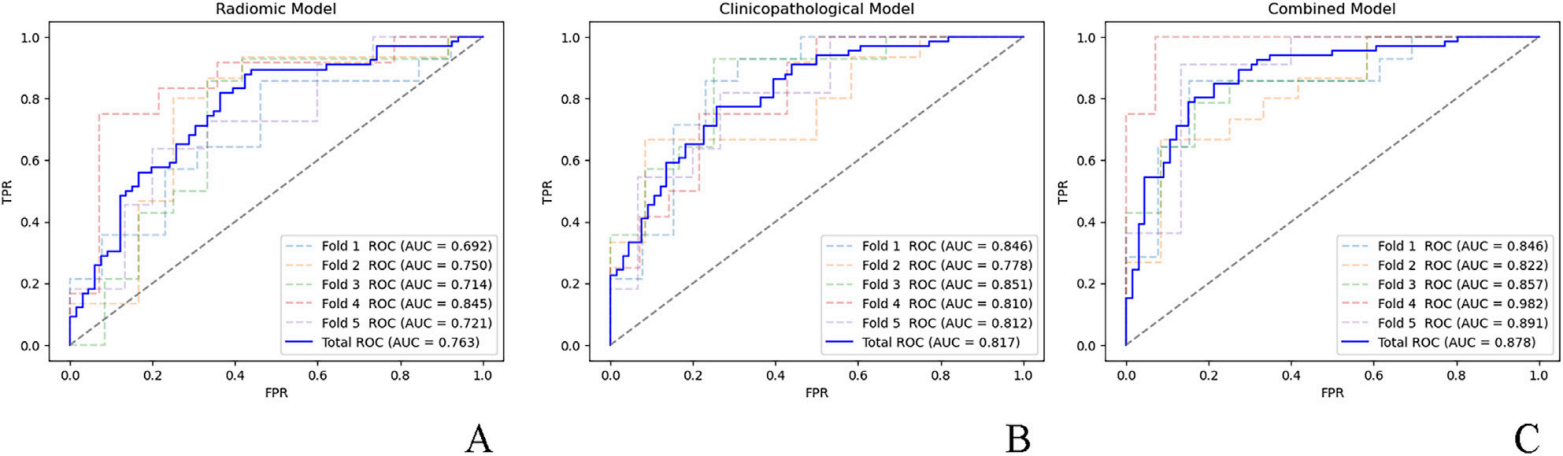
ROC curves for the five-fold cross-validation of the **(A)** radiomic model, **(B)** clinicopathological model, and **(C)** combined model. ROC, receiver operator characteristic; AUC, area under the curve; FPR, false positive rate; TPR, true positive rate.

The ROC curves for the fivefold cross-validation of the models are presented in Figure 1. ROC curve analysis yielded an AUC of 0.763 (95% CI: 0.695–0.836) for the radiomics model and indicated no significant difference (P = 0.36) from the clinicopathological model (AUC = 0.817; 95% CI: 0.781–0.851). The combined model (AUC = 0.878; 95% CI: 0.825–0.973) showed significantly superior performance compared to the clinicopathological (P = 0.02) and radiomic (P < 0.001) models alone. Based on 500 bootstrap iterations, the combined model achieved a mean AUC of 0.879 (SD = 0.030), corresponding to a CV of 0.034, indicating high reproducibility.

As shown in Supplementary Figure S3, there was a weak negative correlation of Rad-score with HLA-I expression (r = −0.31; P < 0.001). No association was found between the Rad-score and other clinicopathological features.

### 3.3 Models for predicting the DFS of patients with *ALK* rearrangement

Among 63 patients with follow-up data, 26 experienced recurrence. The median follow-up period was 53 months, as determined using the reverse Kaplan-Meier method.

For the radiomic model, 400 features were selected by univariate analysis (Supplementary Table S5), and five features with nonzero coefficients remained after LASSO Cox regression (Supplementary Figure S4; Supplementary Table S6). Finally, two independent prognostic features were selected using Cox regression to build the RAD-risk score (Table 3), which was calculated as follows (Equation 3, Supplementary Material 1):

RAD−risk score=0.973×Wavelet_LLH.NGTDM.Busyness+0.566×Wavelet_LLL.GLCM.MaximumProbability (3)

**TABLE 3:** Multivariate Cox regression analyses of radiomic features to predict disease-free survival.

Radiomic feature	Beta value	Hazard ratio (95% CI)	*p*-value
Wavelet_LLH.NGTDM.Busyness	0.973	2.65 (1.78–3.93)	<0.001
Wavelet_LLL.GLCM.MaximumProbability	0.566	1.76 (1.18–2.64)	0.006

Abbreviations: CI, confidence interval; GLCM, gray-level cooccurrence matrix; NGTDM: neighborhood gray tone difference matrix.

**FIGURE 2:**
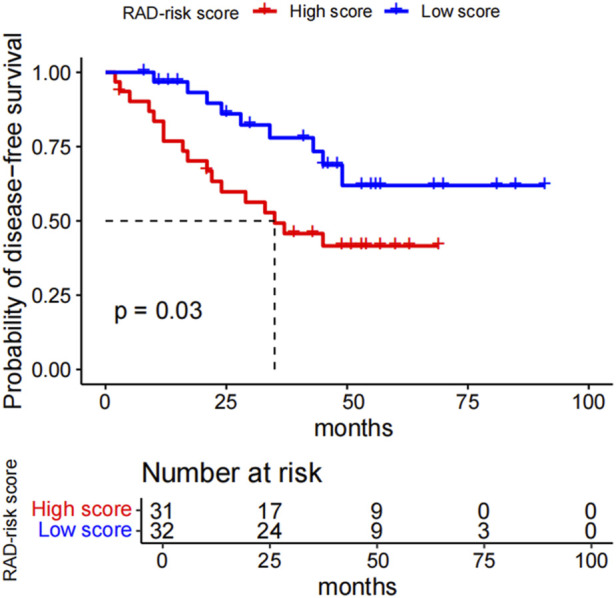
Kaplan-Meier survival curves for the RAD_risk score. With the median RAD_risk score value served as cut-off point, *ALK*-rearranged patients could be divided into two risk groups for disease-free survival (*p* = 0.03). RAD_risk score, radiomics risk score; *ALK*, anaplastic lymphoma kinase.

Kaplan–Meier curves for the dichotomized RAD-risk score are illustrated in Figure 2, which shows that patients with *ALK* rearrangements can be divided into two risk groups. With the median RAD-risk score serving as the cut-off point, a higher RAD-risk score was significantly associated with a lower DFS probability (P = 0.03).

In the clinicopathological model, the univariate analysis showed that sex, T stage, N stage, and pathological stage were significantly associated with DFS (Supplementary Table S7). When these features were included in the multivariate analysis, N stage [hazard ratio (HR) = 5.31; 95% CI: 2.34–12.02; P < 0.001] was an independent predictor of DFS, resulting in a clinicopathological model. Finally, N stage and RAD-risk scores were incorporated into multivariate analysis to develop a combined model (Supplementary Table S8).

The C-indices for each model and 95% CIs are presented in Table 4. There was no significant difference between the radiomic model and the clinicopathological (P > 0.99) or combined models (P = 0.09), while the combined model showed significant improvement (P < 0.001) compared to the clinicopathological model alone. Based on 500 bootstrap iterations, the combined model achieved a mean C-index of 0.811 (SD = 0.042), corresponding to a CV of 0.052, indicating high reproducibility.

**TABLE 4:** Accuracy results of the models for disease-free survival prediction.

Model	C-index	95% CI
Radiomic model	0.752	0.644–0.858
Clinicopathological model	0.712	0.624–0.806
Combined model	0.808	0.723–0.887

Abbreviation: CI, confidence interval.

The RAD-risk score was statistically associated with T stage, N stage, and pathological stage (all P < 0.001) but not with other clinicopathological features (Supplementary Figure S5).

## 4 Discussion

In this study, we developed radiomics, clinicopathological, and combined models for predicting *ALK* rearrangements in lung adenocarcinomas and the DFS of patients with *ALK* rearrangements. The combined models outperformed the clinicopathological models in predicting *ALK* rearrangement status and DFS in patients with *ALK* rearrangement.

Immunosuppressive status is a characteristic of the TIME in patients with *ALK*-rearranged NSCLC (Zhang et al., 2022; Tian et al., 2023). CD8^+^ TILs are reduced in abundance (Zhang et al., 2022; Tian et al., 2023) or functionally impaired despite no decrease in their numbers (Zeng et al., 2020). Several studies have reported an association between PD-L1 expression and *ALK* rearrangements, but with inconsistent results. While Ota *et al.* found that *ALK* rearrangements can upregulate PD-L1 expression in NSCLC (Ota et al., 2015), Zeng *et al.* observed that PD-L1 was seldom expressed in *ALK*-positive tumor cells (Zeng et al., 2020). A meta-analysis concluded that PD-L1 expression did not correlate with *ALK* rearrangement (Zhang et al., 2017). The observed discrepancy in findings may be attributed to the limited sample size of *ALK* rearrangement cases in the studies and differences in ethnicity. We found that PD-L1 and HLA-I expression were adverse predictors of *ALK* rearrangement in lung adenocarcinomas, and that the abundance of CD8^+^ TILs in *ALK*-positive tumors was not different from that in *ALK*-negative tumors. PD-L1 downregulation indicates reduced reliance on the PD-1/PD-L1 axis for immune escape, whereas HLA-I downregulation impairs antigen presentation, rendering CD8^+^ T cells unable to recognize tumor cells. Preserved CD8^+^ T cell density may reflect functional impairment due to T cell exhaustion or antigen recognition impairment. These findings highlight the complexity of immune evasion in *ALK*-rearranged NSCLC and imply the potential involvement of other immune escape mechanisms that require further exploration.

With regard to the radiomic model, we identified four radiomic features as independent predictors of *ALK* rearrangement: one texture feature (Original.GLCM.Autocorrelation), two wavelet-transformed texture features (Wavelet_LLH.GLSZM.SmallAreaEmphasis and Wavelet_HLL.GLCM.Correlation), and one wavelet-transformed texture feature (Wavelet_LLL.Firstorder.Skewness). Skewness, which measures the asymmetry of the histogram from the mean, reflects intratumoral heterogeneity. Texture features are closely associated with tumor heterogeneity and prognosis, whereas wavelet-based features represent filtered transformations of intensity or texture features, capturing multiscale patterns within the tumor (Chen et al., 2017). Due to the low incidence of *ALK*-positive tumors and methodological bias, studies on the association between radiomic features and *ALK* rearrangements in NSCLC remain preliminary (Ninatti et al., 2020; Ma and Li, 2021; Chen et al., 2024). Correlation and skewness (original or filtered) are also found to be predictors of *ALK* rearrangements in previous studies (Agazzi et al., 2021; Choe et al., 2021; Aguloglu et al., 2022; Chen et al., 2025). Interestingly, Wavelet_LLH.GLSZM.SmallAreaEmphasis and Wavelet_LLL.Firstorder.Skewness have been observed to be predictors of brain metastases in patients with *ALK*-rearranged NSCLC (Wang et al., 2022), which may explain the high incidence of brain metastases in patients with *ALK*-rearranged NSCLC. Moreover, Wavelet_LLL.Firstorder.Skewness was also a predictor of DFS in the LASSO Cox regression model (Supplementary Table S5), demonstrating its prognostic value in *ALK*-positive patients.

Regarding prognostic value, PD-L1/HLA-I expression and the density of CD8^+^ TILs were not associated with DFS in patients with *ALK* rearrangement, while the radiomics signature was an independent prognostic factor in our study. Previous studies on the association between PD-L1 expression and progression-free survival (PFS) or overall survival (OS) in patients with *ALK*-rearranged advanced NSCLC treated with *ALK* TKIs found that high PD-L1 expression was associated with shorter PFS or OS (Zhang et al., 2022; Zhou et al., 2022; Tian et al., 2023). Similarly, studies regarding the association between radiomic features and the PFS of patients with *ALK*-rearranged NSCLC were limited to advanced-stage tumors treated with *ALK*-TKIs (Li et al., 2020; Hou et al., 2023; Sun et al., 2023), and also showed the significant prognostic performance of the radiomics signature.

To overcome the limitations of biopsy-related sampling artifacts and ensure robust pathological and molecular data, we focused on surgically resected lung adenocarcinomas. This approach allowed us to investigate the TIME and radiomic features of early stage tumors, offering new insights into their biological characteristics. Our findings may help identify and risk-stratify *ALK*-positive patients at an early stage, inform clinical decision-making, and guide adjuvant therapy or follow-up strategies for high-risk *ALK*-positive patients.

Our study showed that *ALK*-rearranged lung adenocarcinomas exhibit downregulation of PD-L1 and HLA-I, which may contribute to the limited efficacy of PD-1/PD-L1 inhibitors in this population. This observation aligns with emerging clinical evidence showing modest responses to ICIs in *ALK*-positive NSCLC (Mazieres et al., 2019; Jahanzeb et al., 2021). The lack of an association between TIME characteristics and DFS further underscores the need to explore alternative or combination immunotherapeutic strategies, such as targeting innate immune pathways or combining ICIs with *ALK* TKIs. Future studies should investigate these approaches to improve the outcomes for *ALK*-rearranged NSCLC patients.

The radiomics model, when combined with a clinicopathological model incorporating PD-L1 and HLA-I expression to predict *ALK* rearrangement and lymph node metastasis status to predict DFS in *ALK*-rearranged lung adenocarcinomas, demonstrated superior performance compared to the clinicopathological model alone. These findings suggest that radiomic features provide added value for the noninvasive identification of *ALK* rearrangement and prognostic prediction in patients with *ALK*-rearranged lung adenocarcinomas. Notably, we observed a significant correlation between the Rad-score and HLA-I expression as well as between the RAD-risk score and pathological TNM stage. These findings suggest that radiomic features may reflect underlying biological processes, such as immune microenvironment characteristics and tumor progression, thereby enhancing the interpretability of our data-driven models.

This study has several limitations. First, the sample size was relatively small owing to the rarity of *ALK* alterations, which may restrict feature diversity, and the random selection of controls may have introduced a selection bias. Future studies should employ matched designs to reduce the potential confounding factors. Second, as our study focused exclusively on surgically resected lung adenocarcinomas, further studies are needed to validate the applicability of our findings to non-resectable or advanced-stage tumors, which are the primary target population for TKIs or ICIs. Third, the inter-scanner and inter-vendor variability of features may have confounded the results. However, the fact that radiomic features were extracted from multiple scanners in our study may support the generalizability of our models. Moreover, we did not analyze the influence of treatment because only four patients in this early-stage disease cohort received TKI therapy. In future studies, we aim to include a broader patient population, including those receiving TKI therapy, to further evaluate the interplay between treatment, TIME, and outcomes. Finally, although we performed internal cross-validation, multicenter prospective studies with independent external validation are required to confirm our findings.

In conclusion, our results support the potential role of radiomics and TIME in identifying *ALK* rearrangements in lung adenocarcinomas and the prognostic value of radiomics in predicting the DFS of patients with *ALK* rearrangements. We believe that radiomics may improve the risk stratification of patients with *ALK* rearrangements, thereby facilitating personalized treatment. Future studies should validate these models in large multicenter cohorts and integrate multi-omics data (e.g., genomic, transcriptomic, and proteomic) and additional TIME markers to elucidate the biological mechanisms of radiomic features, improve prognostic accuracy, and guide personalized therapy for *ALK*-positive patients.

## Data Availability

The original contributions presented in the study are included in the article/Supplementary Material, further inquiries can be directed to the corresponding author.

## Abbreviations

ALK, anaplastic lymphoma kinase; AUC, area under the curve; CI, confidence interval; CV, coefficient of variation; DFS, disease-free survival; GLCM, gray-level cooccurrence matrix; GLDM, gray-level dependence matrix; GLRLM, gray-level run-length matrix; GLSZM, gray-level size-zone matrix; HLA-I, human leukocyte antigen class I; HR, hazard ratio; ICI, immune checkpoint inhibitor; IHC, immunohistochemistry; LASSO, least absolute shrinkage and selection operator; NGTDM, neighborhood gray-tone difference matrix; NSCLC, non–small cell lung cancer; OR, odds ratio; OS, overall survival; PD-L1, programmed death ligand 1; PFS, progression-free survivall; OC, receiver operator characteristic; TIME, tumor immune microenvironment; TKI, tyrosine kinase inhibitor.
