# Supplementary material for: Radiomic features and tumor immune microenvironment associated with anaplastic lymphoma kinase-rearranged lung adenocarcinoma and their prognostic value
Source: Front Genet. 2025 May 1;16:1581937. doi: 10.3389/fgene.2025.1581937 (PMC12078255; doi:10.3389/fgene.2025.1581937)

Supplementary Figure S1. Flowchart of the patient selection.

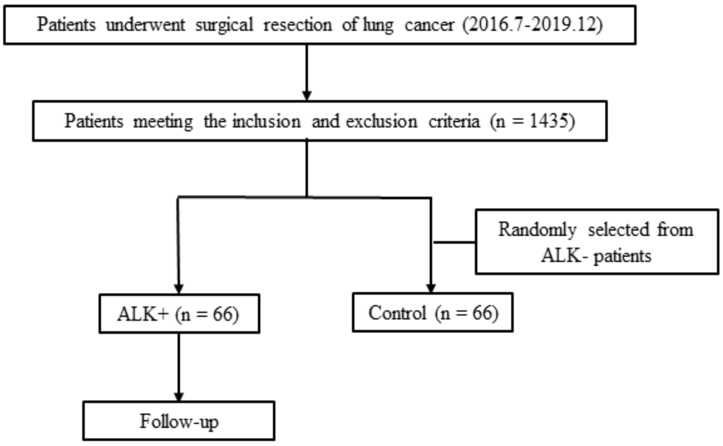

Supplementary Figure S2. Radiomic features selection using the least absolute shrinkage and selection operator (LASSO) logistic regression method. (a) Tuning parameter log (λ) selection in the LASSO model. The dotted vertical line was plotted at the value selected by the 10-fold cross-validation via minimum criteria. (b) LASSO coefficient profiles of the radiomic features. Vertical dashed line represents the optimal λ resulting in non-zero features.

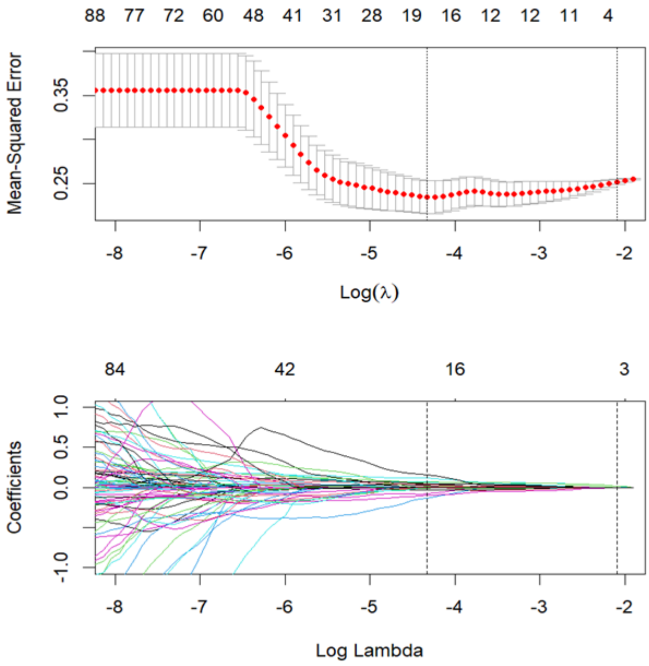

a

b

Supplementary Figure S3. Spearman's correlation coefficient between Rad_score and clinicopathological features

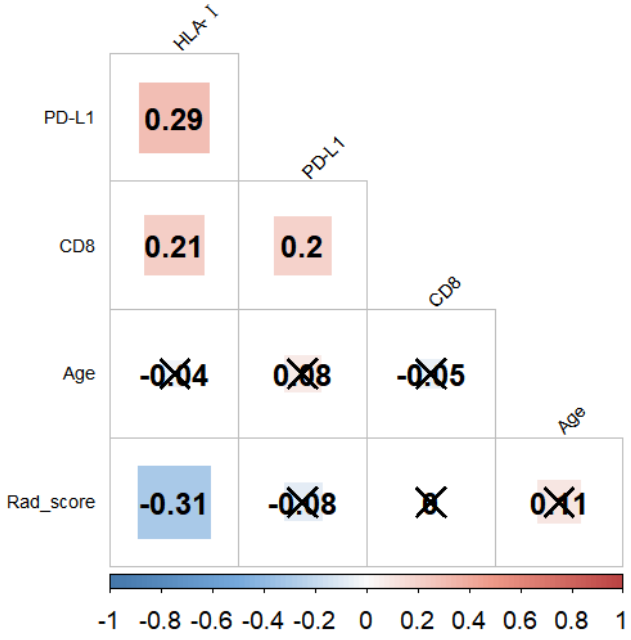

Supplementary Figure S4. Radiomic features selection using the least absolute shrinkage and selection operator (LASSO) Cox regression method. (a) Tuning parameter log (λ) selection in the LASSO model. The dotted vertical line was plotted at the value selected by the 10-fold cross-validation via minimum criteria. (b) LASSO coefficient profiles of the radiomic features. Vertical dashed line represents the optimal λ resulting in non-zero features.

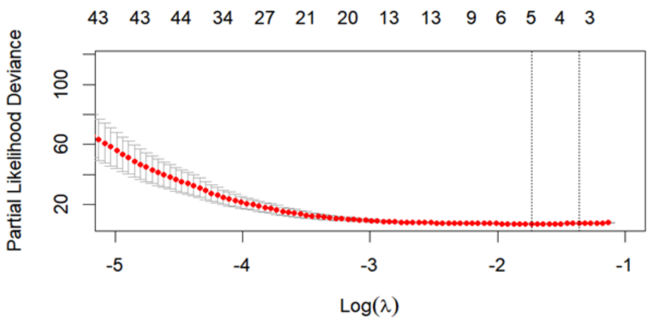

a

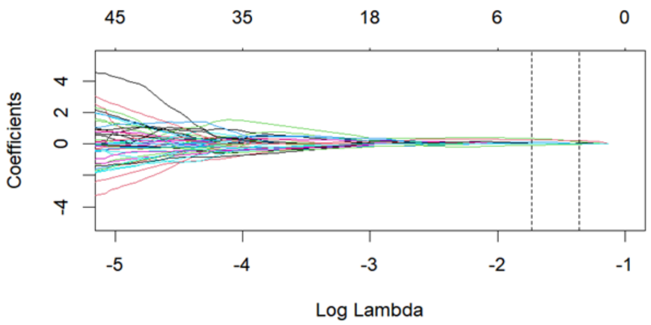

b

Supplementary Figure S5. Association between RAD_risk score and clinicopathological features (***：*P* ＜0.001；　****：*P*＜0.0001)

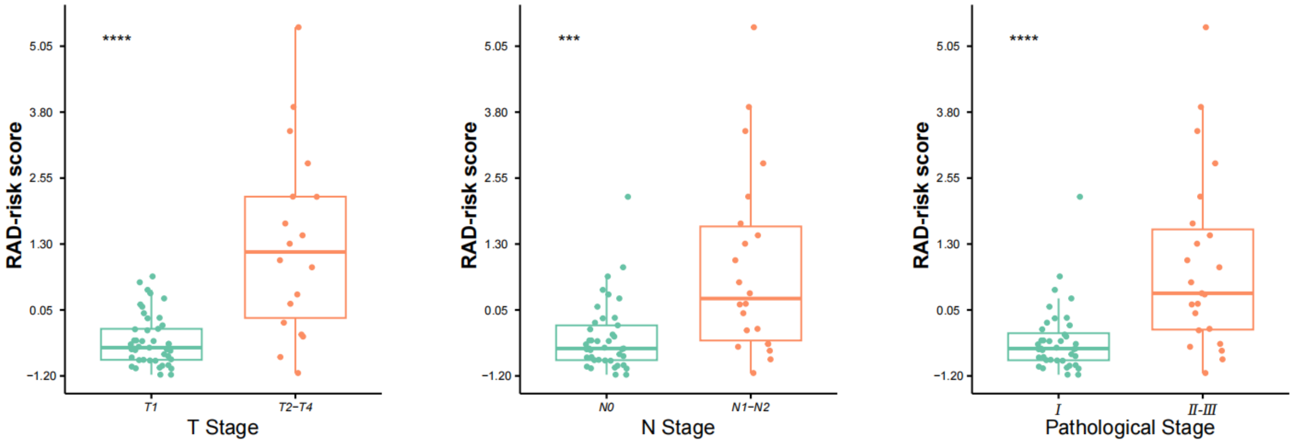

Supplement: Supplementary file 1 [file DataSheet2.pdf]
