# Supplementary material for: Radiomic features and tumor immune microenvironment associated with anaplastic lymphoma kinase-rearranged lung adenocarcinoma and their prognostic value
Source: Front Genet. 2025 May 1;16:1581937. doi: 10.3389/fgene.2025.1581937 (PMC12078255; doi:10.3389/fgene.2025.1581937)
Supplement: Supplementary file 2 [file DataSheet3.pdf]

**Supplementary Table S1 Radiomic features selected by univariate logistic regression**

| | Feature | Odds ratio | 95% CI | *p*-value |
|---|---|---|---|---|
| 1 | originalshapeFlatness | 1.534 | 1.08-2.24 | 0.02 |
| 2 | originalfirstorder90Percentile | 1.684 | 1.16-2.58 | 0.01 |
| 3 | originalfirstorderMean | 1.695 | 1.19-2.48 | 0.005 |
| 4 | originalfirstorderMedian | 1.631 | 1.15-2.36 | 0.008 |
| 5 | originalfirstorderRootMeanSquared | 0.616 | 0.42-0.88 | 0.009 |
| 6 | originalfirstorderSkewness | 0.594 | 0.4-0.85 | 0.006 |
| 7 | originalfirstorderUniformity | 1.510 | 1.04-2.29 | 0.04 |
| 8 | originalglcmAutocorrelation | 1.478 | 1.04-2.14 | 0.03 |
| 9 | originalglcmId | 1.572 | 1.1-2.32 | 0.02 |
| 10 | originalglcmIdm | 1.628 | 1.13-2.43 | 0.01 |
| 11 | originalglcmInverseVariance | 1.576 | 1.1-2.33 | 0.02 |
| 12 | originalglcmJointAverage | 1.531 | 1.08-2.22 | 0.02 |
| 13 | originalglcmJointEnergy | 1.713 | 1.13-2.84 | 0.02 |
| 14 | originalglcmJointEntropy | 0.642 | 0.44-0.92 | 0.02 |
| 15 | originalglcmMCC | 1.448 | 1.02-2.09 | 0.04 |
| 16 | originalglcmMaximumProbability | 1.819 | 1.21-3.02 | 0.009 |
| 17 | originalglcmSumAverage | 1.531 | 1.08-2.22 | 0.02 |
| 18 | originalgldmDependenceNonUniformityNormalized | 0.691 | 0.48-0.98 | 0.04 |
| 19 | originalgldmDependenceVariance | 1.784 | 1.19-2.93 | 0.01 |
| 20 | originalgldmHighGrayLevelEmphasis | 1.464 | 1.03-2.12 | 0.04 |
| 21 | originalgldmLargeDependenceEmphasis | 1.750 | 1.18-2.79 | 0.01 |
| 22 | originalgldmLargeDependenceHighGrayLevelEmphasis | 1.518 | 1.05-2.35 | 0.04 |
| 23 | originalglrlmHighGrayLevelRunEmphasis | 1.462 | 1.03-2.11 | 0.04 |
| 24 | originalglrlmLongRunEmphasis | 1.702 | 1.16-2.65 | 0.01 |
| 25 | originalglrlmLongRunHighGrayLevelEmphasis | 1.523 | 1.07-2.22 | 0.02 |
| 26 | originalglrlmRunLengthNonUniformityNormalized | 0.606 | 0.4-0.88 | 0.01 |
| 27 | originalglrlmRunPercentage | 0.593 | 0.39-0.86 | 0.009 |
| 28 | originalglrlmRunVariance | 1.712 | 1.16-2.71 | 0.01 |
| 29 | originalglrlmShortRunEmphasis | 0.602 | 0.4-0.87 | 0.01 |
| 30 | originalglrlmShortRunHighGrayLevelEmphasis | 1.441 | 1.02-2.08 | 0.04 |
| 31 | originalglszmGrayLevelVariance | 1.454 | 1.03-2.1 | 0.04 |
| 32 | originalglszmSizeZoneNonUniformityNormalized | 1.580 | 1.11-2.32 | 0.02 |
| 33 | originalglszmSmallAreaEmphasis | 1.565 | 1.09-2.31 | 0.02 |
| 34 | originalglszmZonePercentage | 0.683 | 0.47-0.97 | 0.04 |
| 35 | wavelet-LLHfirstorderMaximum | 1.465 | 1.03-2.13 | 0.04 |
| 36 | wavelet-LLHfirstorderRange | 1.566 | 1.08-2.4 | 0.03 |
| 37 | wavelet-LLHglcmMCC | 1.668 | 1.15-2.51 | 0.009 |
| 38 | wavelet-LLHgldmLargeDependenceHighGrayLevelEmphasis | 1.549 | 1.07-2.41 | 0.03 |
| 39 | wavelet-LLHglszmGrayLevelNonUniformityNormalized | 0.598 | 0.4-0.87 | 0.01 |
| 40 | wavelet-LLHglszmGrayLevelVariance | 1.738 | 1.19-2.64 | 0.006 |
| 41 | wavelet-LLHglszmSizeZoneNonUniformityNormalized | 1.892 | 1.3-2.86 | 0.001 |
| 42 | wavelet-LLHglszmSmallAreaEmphasis | 1.919 | 1.31-2.92 | 0.001 |
| 43 | wavelet-LHLfirstorderMean | 0.517 | 0.33-0.77 | 0.002 |
| 44 | wavelet-LHLfirstorderMedian | 0.590 | 0.39-0.86 | 0.008 |
| 45 | wavelet-LHLglcmCorrelation | 1.648 | 1.15-2.43 | 0.008 |

| | | | | |
|---|---|---|---|---|
| 46 | wavelet-LHLglcmId | 1.552 | 1.09-2.28 | 0.02 |
| 47 | wavelet-LHLglcmIdm | 1.563 | 1.09-2.31 | 0.02 |
| 48 | wavelet-LHLglcmInverseVariance | 1.579 | 1.1-2.33 | 0.02 |
| 49 | wavelet-LHLglcmMaximumProbability | 1.565 | 1.08-2.38 | 0.03 |
| 50 | wavelet-LHLgldmDependenceNonUniformityNormalized | 0.651 | 0.45-0.93 | 0.02 |
| 51 | wavelet-LHLgldmDependenceVariance | 1.600 | 1.09-2.49 | 0.02 |
| 52 | wavelet-LHLgldmLargeDependenceEmphasis | 1.580 | 1.09-2.43 | 0.02 |
| 53 | wavelet-LHLgldmSmallDependenceEmphasis | 0.665 | 0.46-0.95 | 0.03 |
| 54 | wavelet-LHLglrlmLongRunEmphasis | 1.577 | 1.09-2.39 | 0.02 |
| 55 | wavelet-LHLglrlmRunLengthNonUniformityNormalized | 0.641 | 0.43-0.92 | 0.02 |
| 56 | wavelet-LHLglrlmRunPercentage | 0.635 | 0.43-0.91 | 0.02 |
| 57 | wavelet-LHLglrlmRunVariance | 1.589 | 1.09-2.43 | 0.02 |
| 58 | wavelet-LHLglrlmShortRunEmphasis | 0.641 | 0.43-0.92 | 0.02 |
| 59 | wavelet-LHLglszmZonePercentage | 0.648 | 0.44-0.92 | 0.02 |
| 60 | wavelet-LHHfirstorderKurtosis | 1.570 | 1.09-2.37 | 0.02 |
| 61 | wavelet-LHHglcmCorrelation | 1.444 | 1.02-2.11 | 0.047 |
| 62 | wavelet-HLLfirstorderInterquartileRange | 0.689 | 0.47-0.98 | 0.045 |
| 63 | wavelet-HLLfirstorderKurtosis | 1.521 | 1.05-2.32 | 0.05 |
| 64 | wavelet-HLLglcmCorrelation | 1.974 | 1.34-3.06 | 0.001 |
| 65 | wavelet-HLLglcmId | 1.566 | 1.09-2.32 | 0.02 |
| 66 | wavelet-HLLglcmIdm | 1.582 | 1.1-2.37 | 0.02 |
| 67 | wavelet-HLLglcmInverseVariance | 1.555 | 1.09-2.3 | 0.02 |
| 68 | wavelet-HLLglcmMaximumProbability | 1.571 | 1.07-2.5 | 0.03 |
| 69 | wavelet-HLLgldmDependenceNonUniformityNormalized | 0.640 | 0.44-0.91 | 0.02 |
| 70 | wavelet-HLLgldmDependenceVariance | 1.552 | 1.06-2.48 | 0.04 |
| 71 | wavelet-HLLgldmLargeDependenceEmphasis | 1.602 | 1.09-2.57 | 0.03 |
| 72 | wavelet-HLLgldmSmallDependenceEmphasis | 0.676 | 0.46-0.96 | 0.03 |
| 73 | wavelet-HLLglrlmLongRunEmphasis | 1.615 | 1.1-2.56 | 0.03 |
| 74 | wavelet-HLLglrlmRunLengthNonUniformityNormalized | 0.614 | 0.4-0.89 | 0.02 |
| 75 | wavelet-HLLglrlmRunPercentage | 0.616 | 0.4-0.9 | 0.02 |
| 76 | wavelet-HLLglrlmRunVariance | 1.593 | 1.08-2.55 | 0.03 |
| 77 | wavelet-HLLglrlmShortRunEmphasis | 0.613 | 0.4-0.89 | 0.02 |
| 78 | wavelet-HLLglszmZonePercentage | 0.657 | 0.45-0.94 | 0.02 |
| 79 | wavelet-HLHfirstorderKurtosis | 1.565 | 1.08-2.4 | 0.03 |
| 80 | wavelet-HLHglcmCorrelation | 1.527 | 1.07-2.22 | 0.02 |
| 81 | wavelet-HHLfirstorderKurtosis | 1.616 | 1.11-2.46 | 0.02 |
| 82 | wavelet-HHLglcmCorrelation | 1.472 | 1.03-2.15 | 0.04 |
| 83 | wavelet-HHLglcmId | 1.447 | 1.02-2.12 | 0.047 |
| 84 | wavelet-HHLglcmIdm | 1.455 | 1.02-2.14 | 0.046 |
| 85 | wavelet-HHLglcmInverseVariance | 1.433 | 1.01-2.08 | 0.049 |
| 86 | wavelet-HHLgldmDependenceNonUniformityNormalized | 0.682 | 0.47-0.97 | 0.04 |
| 87 | wavelet-HHLgldmDependenceVariance | 1.555 | 1.07-2.44 | 0.03 |
| 88 | wavelet-HHLgldmLargeDependenceEmphasis | 1.536 | 1.04-2.46 | 0.047 |
| 89 | wavelet-HHLglrlmLongRunEmphasis | 1.531 | 1.04-2.44 | 0.048 |
| 90 | wavelet-HHLglrlmRunLengthNonUniformityNormalized | 0.666 | 0.45-0.96 | 0.04 |
| 91 | wavelet-HHLglrlmRunPercentage | 0.659 | 0.43-0.95 | 0.04 |
| 92 | wavelet-HHLglrlmRunVariance | 1.542 | 1.04-2.49 | 0.049 |

| | | | | |
|---|---|---|---|---|
| 93 | wavelet-HHLglrlmShortRunEmphasis | 0.665 | 0.44-0.96 | 0.04 |
| 94 | wavelet-HHLglszmZonePercentage | 0.700 | 0.49-0.99 | 0.049 |
| 95 | wavelet-HHHfirstorderKurtosis | 1.550 | 1.08-2.32 | 0.02 |
| 96 | wavelet-LLLfirstorder90Percentile | 1.703 | 1.17-2.62 | 0.009 |
| 97 | wavelet-LLLfirstorderMean | 1.687 | 1.18-2.47 | 0.005 |
| 98 | wavelet-LLLfirstorderMedian | 1.631 | 1.15-2.36 | 0.008 |
| 99 | wavelet-LLLfirstorderRootMeanSquared | 0.618 | 0.42-0.88 | 0.009 |
| 100 | wavelet-LLLfirstorderSkewness | 0.632 | 0.43-0.9 | 0.01 |
| 101 | wavelet-LLLglcmAutocorrelation | 1.483 | 1.05-2.14 | 0.03 |
| 102 | wavelet-LLLglcmDifferenceVariance | 1.558 | 1.09-2.27 | 0.02 |
| 103 | wavelet-LLLglcmIdm | 1.518 | 1.06-2.22 | 0.03 |
| 104 | wavelet-LLLglcmInverseVariance | 1.508 | 1.06-2.2 | 0.03 |
| 105 | wavelet-LLLglcmJointAverage | 1.590 | 1.12-2.31 | 0.01 |
| 106 | wavelet-LLLglcmJointEnergy | 1.578 | 1.07-2.51 | 0.04 |
| 107 | wavelet-LLLglcmMaximumProbability | 1.645 | 1.12-2.55 | 0.02 |
| 108 | wavelet-LLLglcmSumAverage | 1.590 | 1.12-2.31 | 0.01 |
| 109 | wavelet-LLLgldmDependenceVariance | 1.569 | 1.08-2.39 | 0.02 |
| 110 | wavelet-LLLgldmHighGrayLevelEmphasis | 1.468 | 1.04-2.11 | 0.03 |
| 111 | wavelet-LLLgldmLargeDependenceEmphasis | 1.579 | 1.08-2.43 | 0.03 |
| 112 | wavelet-LLLglrlmHighGrayLevelRunEmphasis | 1.468 | 1.04-2.11 | 0.03 |
| 113 | wavelet-LLLglrlmLongRunEmphasis | 1.554 | 1.07-2.35 | 0.03 |
| 114 | wavelet-LLLglrlmLongRunHighGrayLevelEmphasis | 1.480 | 1.04-2.13 | 0.03 |
| 115 | wavelet-LLLglrlmRunLengthNonUniformityNormalized | 0.655 | 0.44-0.94 | 0.03 |
| 116 | wavelet-LLLglrlmRunPercentage | 0.648 | 0.44-0.93 | 0.02 |
| 117 | wavelet-LLLglrlmRunVariance | 1.554 | 1.07-2.37 | 0.03 |
| 118 | wavelet-LLLglrlmShortRunEmphasis | 0.651 | 0.44-0.93 | 0.03 |
| 119 | wavelet-LLLglrlmShortRunHighGrayLevelEmphasis | 1.462 | 1.03-2.11 | 0.04 |
| 120 | wavelet-LLLglszmGrayLevelVariance | 1.532 | 1.08-2.22 | 0.02 |
| 121 | wavelet-LLLglszmHighGrayLevelZoneEmphasis | 1.442 | 1.02-2.08 | 0.04 |
| 122 | wavelet-LLLglszmSmallAreaHighGrayLevelEmphasis | 1.473 | 1.04-2.12 | 0.03 |

**Supplementary Table S2 Radiomic features selected by LASSO regression**

| | **Feature** | **Coefficient** |
|---|---|---|
| 1 | originalshapeFlatness | 0.041 |
| 2 | originalglcmAutocorrelation | -0.006 |
| 3 | originalglcmMCC | 0.019 |
| 4 | originalgldmDependenceVariance | 0.020 |
| 5 | originalgldmLargeDependenceHighGrayLevelEmphasis | -0.131 |
| 6 | originalglszmGrayLevelVariance | -0.078 |
| 7 | wavelet-LLHfirstorderRange | 0.067 |
| 8 | wavelet-LLHglszmGrayLevelNonUniformityNormalized | -0.002 |
| 9 | wavelet-LLHglszmSmallAreaEmphasis | 0.116 |
| 10 | wavelet-LHLfirstorderMean | -0.101 |
| 11 | wavelet-LHLfirstorderMedian | -0.034 |
| 12 | wavelet-HLLfirstorderKurtosis | 0.006 |
| 13 | wavelet-HLLglcmCorrelation | 0.066 |
| 14 | wavelet-HLHglcmCorrelation | 0.047 |
| 15 | wavelet-HHLfirstorderKurtosis | 0.033 |
| 16 | wavelet-LLLfirstorderSkewness | -0.012 |
| 17 | wavelet-LLLglcmInverseVariance | 0.157 |
| | Intercept | 0.500 |

**Supplementary Table S3 Univariate logistic regression analysis of clinicopathological features**

| Feature | Odds ratio | 95% CI | *p*-value |
|---|---|---|---|
| HLA-I | 0.986 | 0.98-0.99 | **<0.001** |
| PD-L1 | 0.987 | 0.98-0.99 | **<0.001** |
| CD8 | 1.012 | 0.97-1.06 | 0.60 |
| Sex | 0.738 | 0.37-1.46 | 0.38 |
| Age | 0.996 | 0.96-1.03 | 0.81 |
| Smoking History | 0.531 | 0.26-1.07 | 0.08 |
| T Stage | 1.000 | 0.46-2.16 | >0.99 |
| N Stage | 1.562 | 0.73-3.38 | 0.25 |
| Pathological Stage | 1.323 | 0.64-2.78 | 0.46 |

Bolded values indicate a statistically significant result.

**Supplementary Table S4 Multivariate regression analyses of clinicopathological features and Rad-score**

| Feature | Odds ratio | 95% CI | *p*-value |
|---|---|---|---|
| HLA-I | 0.99 | 0.98-0.99 | <0.001 |
| PD-L1 | 0.99 | 0.98-0.99 | <0.001 |
| Rad-score | 2.88 | 1.82-4.88 | <0.001 |

**Supplementary Table S5 Radiomic features selected by univariate Cox regression**

| | Feature | Hazard ratio | 95% CI | *p*-value |
|---|---|---|---|---|
| 1 | originalshapeLeastAxisLength | 2.860 | 1.88- 4.35 | <0.001 |
| 2 | originalshapeMajorAxisLength | 1.690 | 1.19-2.4 | 0.003 |
| 3 | originalshapeMaximum2DDiameterColumn | 2.230 | 1.56-3.19 | <0.001 |
| 4 | originalshapeMaximum2DDiameterRow | 2.040 | 1.45-2.87 | <0.001 |
| 5 | originalshapeMeshVolume | 2.380 | 1.73-3.27 | <0.001 |
| 6 | originalshapeMinorAxisLength | 2.430 | 1.67-3.53 | <0.001 |
| 7 | originalshapeSurfaceArea | 2.380 | 1.7-3.33 | <0.001 |
| 8 | originalshapeSurfaceVolumeRatio | 0.310 | 0.16-0.59 | <0.001 |
| 9 | originalshapeVoxelVolume | 2.380 | 1.73-3.27 | <0.001 |
| 10 | originalfirstorder10Percentile | 2.760 | 1.83-4.17 | <0.001 |
| 11 | originalfirstorder90Percentile | 2.980 | 1.29-6.86 | 0.01 |
| 12 | originalfirstorderEnergy | 1.940 | 1.28-2.94 | 0.002 |
| 13 | originalfirstorderEntropy | 0.450 | 0.29-0.69 | <0.001 |
| 14 | originalfirstorderInterquartileRange | 0.590 | 0.38-0.92 | 0.02 |
| 15 | originalfirstorderMeanAbsoluteDeviation | 0.520 | 0.32-0.86 | 0.01 |
| 16 | originalfirstorderMean | 2.930 | 1.62-5.31 | <0.001 |
| 17 | originalfirstorderMedian | 2.660 | 1.42-4.98 | 0.002 |
| 18 | originalfirstorderRobustMeanAbsoluteDeviation | 0.560 | 0.35-0.89 | 0.01 |
| 19 | originalfirstorderRootMeanSquared | 0.290 | 0.16-0.54 | <0.001 |
| 20 | originalfirstorderSkewness | 0.340 | 0.2-0.58 | <0.001 |
| 21 | originalfirstorderTotalEnergy | 1.850 | 1.26-2.73 | 0.002 |
| 22 | originalfirstorderUniformity | 2.340 | 1.55-3.55 | <0.001 |
| 23 | originalfirstorderVariance | 0.590 | 0.35-0.99 | 0.045 |
| 24 | originalglcmAutocorrelation | 2.100 | 1.36-3.26 | 0.001 |
| 25 | originalglcmClusterTendency | 0.570 | 0.33-0.99 | 0.045 |
| 26 | originalglcmContrast | 0.570 | 0.34-0.93 | 0.03 |
| 27 | originalglcmDifferenceAverage | 0.540 | 0.34-0.85 | 0.008 |
| 28 | originalglcmDifferenceEntropy | 0.510 | 0.33-0.79 | 0.002 |
| 29 | originalglcmId | 1.750 | 1.15-2.67 | 0.009 |
| 30 | originalglcmIdm | 1.710 | 1.13-2.6 | 0.01 |
| 31 | originalglcmIdmn | 1.980 | 1.27-3.1 | 0.003 |
| 32 | originalglcmIdn | 1.960 | 1.28-3 | 0.002 |
| 33 | originalglcmImc1 | 2.690 | 1.19-6.07 | 0.02 |
| 34 | originalglcmImc2 | 0.520 | 0.37-0.74 | <0.001 |
| 35 | originalglcmInverseVariance | 1.750 | 1.15-2.64 | 0.009 |
| 36 | originalglcmJointAverage | 2.430 | 1.44-4.11 | 0.001 |
| 37 | originalglcmJointEnergy | 2.120 | 1.41-3.19 | <0.001 |
| 38 | originalglcmJointEntropy | 0.610 | 0.4-0.94 | 0.02 |
| 39 | originalglcmSumAverage | 2.430 | 1.44-4.11 | 0.001 |
| 40 | originalglcmSumEntropy | 0.440 | 0.28-0.68 | <0.001 |
| 41 | originalglcmSumSquares | 0.540 | 0.3-0.96 | 0.03 |
| 42 | originalgldmDependenceNonUniformity | 1.980 | 1.5-2.61 | <0.001 |
| 43 | originalgldmDependenceNonUniformityNormalized | 0.570 | 0.35-0.91 | 0.02 |
| 44 | originalgldmGrayLevelNonUniformity | 2.860 | 1.98-4.13 | <0.001 |
| 45 | originalgldmGrayLevelVariance | 0.590 | 0.35-0.99 | 0.045 |

| | | | | |
|---|---|---|---|---|
| 46 | originalgldmHighGrayLevelEmphasis | 2.260 | 1.42-3.58 | 0.001 |
| 47 | originalgldmLargeDependenceEmphasis | 1.520 | 1.05-2.19 | 0.03 |
| 48 | originalgldmLargeDependenceHighGrayLevelEmphasis | 1.620 | 1.17-2.25 | 0.004 |
| 49 | originalgldmSmallDependenceEmphasis | 0.530 | 0.34-0.83 | 0.005 |
| 50 | originalgldmSmallDependenceHighGrayLevelEmphasis | 1.480 | 1.05-2.09 | 0.03 |
| 51 | originalgldmSmallDependenceLowGrayLevelEmphasis | 0.360 | 0.2-0.65 | 0.001 |
| 52 | originalglrlmGrayLevelNonUniformity | 2.900 | 1.99-4.23 | <0.001 |
| 53 | originalglrlmGrayLevelNonUniformityNormalized | 2.290 | 1.53-3.44 | <0.001 |
| 54 | originalglrlmHighGrayLevelRunEmphasis | 2.310 | 1.44-3.69 | <0.001 |
| 55 | originalglrlmLongRunEmphasis | 1.700 | 1.15-2.52 | 0.008 |
| 56 | originalglrlmLongRunHighGrayLevelEmphasis | 2.150 | 1.44-3.21 | <0.001 |
| 57 | originalglrlmRunEntropy | 0.480 | 0.31-0.75 | 0.001 |
| 58 | originalglrlmRunLengthNonUniformity | 2.210 | 1.64-2.99 | <0.001 |
| 59 | originalglrlmRunLengthNonUniformityNormalized | 0.540 | 0.35-0.83 | 0.005 |
| 60 | originalglrlmRunPercentage | 0.570 | 0.38-0.86 | 0.008 |
| 61 | originalglrlmRunVariance | 1.540 | 1.07-2.22 | 0.02 |
| 62 | originalglrlmShortRunEmphasis | 0.530 | 0.35-0.82 | 0.004 |
| 63 | originalglrlmShortRunHighGrayLevelEmphasis | 2.280 | 1.42-3.64 | 0.001 |
| 64 | originalglrlmShortRunLowGrayLevelEmphasis | 0.490 | 0.26-0.93 | 0.03 |
| 65 | originalglszmGrayLevelNonUniformity | 2.010 | 1.53-2.65 | <0.001 |
| 66 | originalglszmGrayLevelNonUniformityNormalized | 1.790 | 1.13-2.83 | 0.01 |
| 67 | originalglszmHighGrayLevelZoneEmphasis | 2.300 | 1.49-3.54 | <0.001 |
| 68 | originalglszmLargeAreaEmphasis | 2.340 | 1.56-3.51 | <0.001 |
| 69 | originalglszmLargeAreaHighGrayLevelEmphasis | 2.340 | 1.56-3.5 | <0.001 |
| 70 | originalglszmLowGrayLevelZoneEmphasis | 0.460 | 0.28-0.76 | 0.002 |
| 71 | originalglszmSizeZoneNonUniformity | 2.000 | 1.51-2.64 | <0.001 |
| 72 | originalglszmSmallAreaHighGrayLevelEmphasis | 2.150 | 1.42-3.26 | <0.001 |
| 73 | originalglszmSmallAreaLowGrayLevelEmphasis | 0.430 | 0.26-0.71 | 0.001 |
| 74 | originalglszmZonePercentage | 0.530 | 0.34-0.82 | 0.004 |
| 75 | originalglszmZoneVariance | 2.340 | 1.56-3.51 | <0.001 |
| 76 | originalngtdmBusyness | 2.570 | 1.75-3.77 | <0.001 |
| 77 | originalngtdmContrast | 0.490 | 0.29-0.83 | 0.008 |
| 78 | originalngtdmStrength | 0.340 | 0.12-0.97 | 0.04 |
| 79 | wavelet-LLHfirstorder10Percentile | 2.250 | 1.34-3.76 | 0.002 |
| 80 | wavelet-LLHfirstorder90Percentile | 0.480 | 0.29-0.81 | 0.006 |
| 81 | wavelet-LLHfirstorderEnergy | 2.230 | 1.57-3.18 | <0.001 |
| 82 | wavelet-LLHfirstorderEntropy | 0.390 | 0.24-0.65 | <0.001 |
| 83 | wavelet-LLHfirstorderInterquartileRange | 0.520 | 0.32-0.85 | 0.009 |
| 84 | wavelet-LLHfirstorderMeanAbsoluteDeviation | 0.450 | 0.26-0.77 | 0.004 |
| 85 | wavelet-LLHfirstorderRobustMeanAbsoluteDeviation | 0.490 | 0.3-0.81 | 0.005 |
| 86 | wavelet-LLHfirstorderRootMeanSquared | 0.470 | 0.28-0.79 | 0.005 |
| 87 | wavelet-LLHfirstorderSkewness | 1.920 | 1.15-3.19 | 0.01 |
| 88 | wavelet-LLHfirstorderTotalEnergy | 2.100 | 1.51-2.92 | <0.001 |
| 89 | wavelet-LLHfirstorderUniformity | 2.670 | 1.72-4.14 | <0.001 |
| 90 | wavelet-LLHfirstorderVariance | 0.500 | 0.29-0.88 | 0.02 |
| 91 | wavelet-LLHglcmClusterProminence | 0.490 | 0.24-0.99 | 0.047 |
| 92 | wavelet-LLHglcmClusterTendency | 0.460 | 0.25-0.84 | 0.01 |

| | | | | |
|---|---|---|---|---|
| 93 | wavelet-LLHglcmContrast | 0.480 | 0.26-0.87 | 0.02 |
| 94 | wavelet-LLHglcmDifferenceAverage | 0.450 | 0.26-0.78 | 0.004 |
| 95 | wavelet-LLHglcmDifferenceEntropy | 0.410 | 0.25-0.68 | 0.001 |
| 96 | wavelet-LLHglcmDifferenceVariance | 0.520 | 0.3-0.92 | 0.03 |
| 97 | wavelet-LLHglcmId | 2.220 | 1.41-3.52 | 0.001 |
| 98 | wavelet-LLHglcmIdm | 2.200 | 1.41-3.44 | 0.001 |
| 99 | wavelet-LLHglcmIdmn | 2.230 | 1.24-4.02 | 0.007 |
| 100 | wavelet-LLHglcmIdn | 2.070 | 1.29-3.32 | 0.003 |
| 101 | wavelet-LLHglcmImc2 | 0.580 | 0.39-0.87 | 0.009 |
| 102 | wavelet-LLHglcmInverseVariance | 2.150 | 1.38-3.37 | 0.001 |
| 103 | wavelet-LLHglcmJointEnergy | 2.710 | 1.73-4.24 | <0.001 |
| 104 | wavelet-LLHglcmJointEntropy | 0.520 | 0.32-0.84 | 0.007 |
| 105 | wavelet-LLHglcmMaximumProbability | 2.130 | 1.42-3.18 | <0.001 |
| 106 | wavelet-LLHglcmSumEntropy | 0.380 | 0.23-0.62 | <0.001 |
| 107 | wavelet-LLHglcmSumSquares | 0.470 | 0.26-0.85 | 0.01 |
| 108 | wavelet-LLHgldmDependenceNonUniformity | 2.220 | 1.63-3.02 | <0.001 |
| 109 | wavelet-LLHgldmDependenceNonUniformityNormalized | 0.480 | 0.29-0.78 | 0.003 |
| 110 | wavelet-LLHgldmDependenceVariance | 1.800 | 1.27-2.55 | 0.001 |
| 111 | wavelet-LLHgldmGrayLevelNonUniformity | 2.620 | 1.85-3.71 | <0.001 |
| 112 | wavelet-LLHgldmGrayLevelVariance | 0.500 | 0.29-0.88 | 0.02 |
| 113 | wavelet-LLHgldmLargeDependenceEmphasis | 2.330 | 1.56-3.48 | <0.001 |
| 114 | wavelet-LLHgldmLargeDependenceHighGrayLevelEmphasis | 1.410 | 1.05-1.88 | 0.02 |
| 115 | wavelet-LLHgldmSmallDependenceEmphasis | 0.430 | 0.26-0.72 | 0.001 |
| 116 | wavelet-LLHglrlmGrayLevelNonUniformity | 2.510 | 1.79-3.5 | <0.001 |
| 117 | wavelet-LLHglrlmGrayLevelNonUniformityNormalized | 2.650 | 1.7-4.14 | <0.001 |
| 118 | wavelet-LLHglrlmGrayLevelVariance | 0.520 | 0.3-0.89 | 0.02 |
| 119 | wavelet-LLHglrlmLongRunEmphasis | 2.460 | 1.62-3.75 | <0.001 |
| 120 | wavelet-LLHglrlmRunEntropy | 0.470 | 0.29-0.78 | 0.004 |
| 121 | wavelet-LLHglrlmRunLengthNonUniformity | 2.290 | 1.68-3.13 | <0.001 |
| 122 | wavelet-LLHglrlmRunLengthNonUniformityNormalized | 0.420 | 0.27-0.66 | <0.001 |
| 123 | wavelet-LLHglrlmRunPercentage | 0.420 | 0.27-0.65 | <0.001 |
| 124 | wavelet-LLHglrlmRunVariance | 2.380 | 1.59-3.56 | <0.001 |
| 125 | wavelet-LLHglrlmShortRunEmphasis | 0.410 | 0.26-0.64 | <0.001 |
| 126 | wavelet-LLHglszmGrayLevelNonUniformity | 2.250 | 1.66-3.06 | <0.001 |
| 127 | wavelet-LLHglszmGrayLevelNonUniformityNormalized | 1.770 | 1.14-2.73 | 0.01 |
| 128 | wavelet-LLHglszmLargeAreaEmphasis | 3.220 | 1.97-5.28 | <0.001 |
| 129 | wavelet-LLHglszmLargeAreaHighGrayLevelEmphasis | 1.720 | 1.34-2.21 | <0.001 |
| 130 | wavelet-LLHglszmLargeAreaLowGrayLevelEmphasis | 5.510 | 2.46-12.37 | <0.001 |
| 131 | wavelet-LLHglszmSizeZoneNonUniformity | 2.330 | 1.68-3.24 | <0.001 |
| 132 | wavelet-LLHglszmZonePercentage | 0.430 | 0.26-0.7 | 0.001 |
| 133 | wavelet-LLHglszmZoneVariance | 3.220 | 1.97-5.28 | <0.001 |
| 134 | wavelet-LLHngtdmBusyness | 2.950 | 2.05-4.24 | <0.001 |
| 135 | wavelet-LLHngtdmContrast | 0.420 | 0.2-0.87 | 0.02 |
| 136 | wavelet-LLHngtdmStrength | 0.280 | 0.09-0.9 | 0.03 |
| 137 | wavelet-LHLfirstorderEnergy | 1.780 | 1.36-2.33 | <0.001 |
| 138 | wavelet-LHLfirstorderKurtosis | 1.500 | 1.03-2.17 | 0.03 |
| 139 | wavelet-LHLfirstorderMaximum | 1.760 | 1.2-2.59 | 0.004 |

| | | | | |
|---|---|---|---|---|
| 140 | wavelet-LHLfirstorderRange | 1.660 | 1.12-2.48 | 0.01 |
| 141 | wavelet-LHLfirstorderSkewness | 2.350 | 1.51-3.67 | <0.001 |
| 142 | wavelet-LHLfirstorderTotalEnergy | 1.740 | 1.34-2.25 | <0.001 |
| 143 | wavelet-LHLglcmAutocorrelation | 1.550 | 1.1-2.19 | 0.01 |
| 144 | wavelet-LHLglcmCorrelation | 0.640 | 0.44-0.92 | 0.02 |
| 145 | wavelet-LHLglcmIdmn | 2.020 | 1.12-3.64 | 0.02 |
| 146 | wavelet-LHLglcmIdn | 1.900 | 1.15-3.12 | 0.01 |
| 147 | wavelet-LHLglcmImc2 | 0.600 | 0.39-0.92 | 0.02 |
| 148 | wavelet-LHLglcmJointAverage | 1.510 | 1-2.26 | 0.049 |
| 149 | wavelet-LHLglcmSumAverage | 1.510 | 1-2.26 | 0.049 |
| 150 | wavelet-LHLgldmDependenceNonUniformity | 1.920 | 1.47-2.51 | <0.001 |
| 151 | wavelet-LHLgldmGrayLevelNonUniformity | 2.070 | 1.57-2.73 | <0.001 |
| 152 | wavelet-LHLgldmHighGrayLevelEmphasis | 1.550 | 1.09-2.19 | 0.01 |
| 153 | wavelet-LHLgldmLargeDependenceHighGrayLevelEmphasis | 1.880 | 1.31-2.69 | 0.001 |
| 154 | wavelet-LHLglrlmGrayLevelNonUniformity | 2.150 | 1.61-2.86 | <0.001 |
| 155 | wavelet-LHLglrlmHighGrayLevelRunEmphasis | 1.550 | 1.09-2.19 | 0.01 |
| 156 | wavelet-LHLglrlmLongRunHighGrayLevelEmphasis | 1.600 | 1.14-2.26 | 0.007 |
| 157 | wavelet-LHLglrlmRunLengthNonUniformity | 2.240 | 1.66-3.04 | <0.001 |
| 158 | wavelet-LHLglrlmShortRunHighGrayLevelEmphasis | 1.530 | 1.08-2.17 | 0.02 |
| 159 | wavelet-LHLglszmGrayLevelNonUniformity | 2.140 | 1.6-2.87 | <0.001 |
| 160 | wavelet-LHLglszmHighGrayLevelZoneEmphasis | 1.550 | 1.1-2.19 | 0.01 |
| 161 | wavelet-LHLglszmLargeAreaEmphasis | 1.600 | 1.24-2.07 | <0.001 |
| 162 | wavelet-LHLglszmLargeAreaHighGrayLevelEmphasis | 1.890 | 1.4-2.54 | <0.001 |
| 163 | wavelet-LHLglszmLargeAreaLowGrayLevelEmphasis | 1.470 | 1.14-1.9 | 0.003 |
| 164 | wavelet-LHLglszmSizeZoneNonUniformity | 1.880 | 1.44-2.45 | <0.001 |
| 165 | wavelet-LHLglszmSmallAreaHighGrayLevelEmphasis | 1.540 | 1.09-2.17 | 0.02 |
| 166 | wavelet-LHLglszmZoneVariance | 1.600 | 1.24-2.07 | <0.001 |
| 167 | wavelet-LHLngtdmBusyness | 2.130 | 1.55-2.93 | <0.001 |
| 168 | wavelet-LHLngtdmStrength | 0.520 | 0.3-0.93 | 0.03 |
| 169 | wavelet-LHHfirstorderEnergy | 1.600 | 1.25-2.05 | <0.001 |
| 170 | wavelet-LHHfirstorderMaximum | 1.590 | 1.1-2.3 | 0.01 |
| 171 | wavelet-LHHfirstorderRange | 1.530 | 1.05-2.22 | 0.03 |
| 172 | wavelet-LHHfirstorderTotalEnergy | 1.580 | 1.24-2.02 | <0.001 |
| 173 | wavelet-LHHglcmIdn | 1.640 | 1.01-2.65 | 0.04 |
| 174 | wavelet-LHHgldmDependenceNonUniformity | 1.930 | 1.47-2.52 | <0.001 |
| 175 | wavelet-LHHgldmGrayLevelNonUniformity | 1.970 | 1.51-2.56 | <0.001 |
| 176 | wavelet-LHHgldmLargeDependenceHighGrayLevelEmphasis | 2.030 | 1.39-2.98 | <0.001 |
| 177 | wavelet-LHHgldmLowGrayLevelEmphasis | 0.470 | 0.22-0.98 | 0.04 |
| 178 | wavelet-LHHglrlmGrayLevelNonUniformity | 2.090 | 1.58-2.77 | <0.001 |
| 179 | wavelet-LHHglrlmLongRunHighGrayLevelEmphasis | 1.440 | 1.04-2 | 0.03 |
| 180 | wavelet-LHHglrlmLongRunLowGrayLevelEmphasis | 0.510 | 0.26-1 | 0.049 |
| 181 | wavelet-LHHglrlmLowGrayLevelRunEmphasis | 0.470 | 0.22-0.98 | 0.045 |
| 182 | wavelet-LHHglrlmRunLengthNonUniformity | 2.210 | 1.64-2.97 | <0.001 |
| 183 | wavelet-LHHglrlmShortRunLowGrayLevelEmphasis | 0.460 | 0.21-0.98 | 0.045 |
| 184 | wavelet-LHHglszmGrayLevelNonUniformity | 2.120 | 1.59-2.83 | <0.001 |
| 185 | wavelet-LHHglszmLargeAreaEmphasis | 1.510 | 1.18-1.93 | 0.001 |
| 186 | wavelet-LHHglszmLargeAreaHighGrayLevelEmphasis | 1.660 | 1.27-2.16 | <0.001 |

| | | | | |
|---|---|---|---|---|
| 187 | wavelet-LHHglszmLowGrayLevelZoneEmphasis | 0.470 | 0.22-1 | 0.049 |
| 188 | wavelet-LHHglszmSizeZoneNonUniformity | 1.860 | 1.43-2.43 | <0.001 |
| 189 | wavelet-LHHglszmZoneVariance | 1.510 | 1.18-1.93 | 0.001 |
| 190 | wavelet-LHHngtdmBusyness | 2.280 | 1.63-3.18 | <0.001 |
| 191 | wavelet-LHHngtdmStrength | 0.460 | 0.22-0.97 | 0.04 |
| 192 | wavelet-HLLfirstorder10Percentile | 1.560 | 1.02-2.36 | 0.04 |
| 193 | wavelet-HLLfirstorder90Percentile | 0.610 | 0.4-0.94 | 0.02 |
| 194 | wavelet-HLLfirstorderEnergy | 2.020 | 1.46-2.78 | <0.001 |
| 195 | wavelet-HLLfirstorderEntropy | 0.630 | 0.44-0.91 | 0.01 |
| 196 | wavelet-HLLfirstorderInterquartileRange | 0.630 | 0.41-0.95 | 0.03 |
| 197 | wavelet-HLLfirstorderMeanAbsoluteDeviation | 0.640 | 0.43-0.95 | 0.03 |
| 198 | wavelet-HLLfirstorderRobustMeanAbsoluteDeviation | 0.620 | 0.41-0.94 | 0.02 |
| 199 | wavelet-HLLfirstorderTotalEnergy | 1.960 | 1.44-2.65 | <0.001 |
| 200 | wavelet-HLLfirstorderUniformity | 1.860 | 1.29-2.7 | 0.001 |
| 201 | wavelet-HLLglcmClusterTendency | 0.600 | 0.38-0.96 | 0.03 |
| 202 | wavelet-HLLglcmDifferenceAverage | 0.660 | 0.45-0.98 | 0.04 |
| 203 | wavelet-HLLglcmDifferenceEntropy | 0.680 | 0.48-0.97 | 0.03 |
| 204 | wavelet-HLLglcmId | 1.560 | 1.1-2.24 | 0.01 |
| 205 | wavelet-HLLglcmIdm | 1.570 | 1.11-2.24 | 0.01 |
| 206 | wavelet-HLLglcmIdmn | 1.750 | 1.05-2.93 | 0.03 |
| 207 | wavelet-HLLglcmIdn | 1.690 | 1.08-2.63 | 0.02 |
| 208 | wavelet-HLLglcmImc2 | 0.580 | 0.38-0.87 | 0.009 |
| 209 | wavelet-HLLglcmInverseVariance | 1.600 | 1.1-2.31 | 0.01 |
| 210 | wavelet-HLLglcmJointEnergy | 2.020 | 1.39-2.96 | <0.001 |
| 211 | wavelet-HLLglcmMaximumProbability | 1.550 | 1.12-2.16 | 0.009 |
| 212 | wavelet-HLLglcmSumEntropy | 0.520 | 0.35-0.79 | 0.002 |
| 213 | wavelet-HLLglcmSumSquares | 0.630 | 0.4-0.99 | 0.045 |
| 214 | wavelet-HLLgldmDependenceNonUniformity | 1.970 | 1.49-2.6 | <0.001 |
| 215 | wavelet-HLLgldmDependenceNonUniformityNormalized | 0.570 | 0.37-0.87 | 0.01 |
| 216 | wavelet-HLLgldmDependenceVariance | 1.440 | 1.08-1.93 | 0.01 |
| 217 | wavelet-HLLgldmGrayLevelNonUniformity | 2.440 | 1.78-3.34 | <0.001 |
| 218 | wavelet-HLLgldmLargeDependenceEmphasis | 1.620 | 1.18-2.23 | 0.003 |
| 219 | wavelet-HLLgldmLargeDependenceHighGrayLevelEmphasis | 1.670 | 1.12-2.5 | 0.01 |
| 220 | wavelet-HLLgldmSmallDependenceEmphasis | 0.620 | 0.43-0.9 | 0.01 |
| 221 | wavelet-HLLglrlmGrayLevelNonUniformity | 2.420 | 1.76-3.32 | <0.001 |
| 222 | wavelet-HLLglrlmGrayLevelNonUniformityNormalized | 1.770 | 1.23-2.57 | 0.002 |
| 223 | wavelet-HLLglrlmLongRunEmphasis | 1.700 | 1.22-2.38 | 0.002 |
| 224 | wavelet-HLLglrlmRunLengthNonUniformity | 2.200 | 1.63-2.97 | <0.001 |
| 225 | wavelet-HLLglrlmRunLengthNonUniformityNormalized | 0.580 | 0.4-0.82 | 0.002 |
| 226 | wavelet-HLLglrlmRunPercentage | 0.580 | 0.41-0.83 | 0.002 |
| 227 | wavelet-HLLglrlmRunVariance | 1.650 | 1.19-2.27 | 0.002 |
| 228 | wavelet-HLLglrlmShortRunEmphasis | 0.570 | 0.4-0.81 | 0.002 |
| 229 | wavelet-HLLglszmGrayLevelNonUniformity | 2.040 | 1.54-2.71 | <0.001 |
| 230 | wavelet-HLLglszmLargeAreaEmphasis | 2.430 | 1.57-3.75 | <0.001 |
| 231 | wavelet-HLLglszmLargeAreaHighGrayLevelEmphasis | 2.540 | 1.68-3.84 | <0.001 |
| 232 | wavelet-HLLglszmLargeAreaLowGrayLevelEmphasis | 2.030 | 1.39-2.97 | <0.001 |
| 233 | wavelet-HLLglszmSizeZoneNonUniformity | 1.980 | 1.49-2.63 | <0.001 |

| | | | | |
|---|---|---|---|---|
| 234 | wavelet-HLLglszmZonePercentage | 0.600 | 0.41-0.88 | 0.008 |
| 235 | wavelet-HLLglszmZoneVariance | 2.430 | 1.57-3.75 | <0.001 |
| 236 | wavelet-HLLngtdmBusyness | 2.240 | 1.62-3.09 | <0.001 |
| 237 | wavelet-HLLngtdmContrast | 0.490 | 0.28-0.88 | 0.02 |
| 238 | wavelet-HLLngtdmStrength | 0.340 | 0.16-0.72 | 0.005 |
| 239 | wavelet-HLHfirstorderEnergy | 2.010 | 1.49-2.7 | <0.001 |
| 240 | wavelet-HLHfirstorderKurtosis | 1.610 | 1.08-2.4 | 0.02 |
| 241 | wavelet-HLHfirstorderTotalEnergy | 1.980 | 1.48-2.65 | <0.001 |
| 242 | wavelet-HLHglcmIdmn | 1.850 | 1.08-3.14 | 0.02 |
| 243 | wavelet-HLHglcmIdn | 1.760 | 1.12-2.79 | 0.02 |
| 244 | wavelet-HLHglcmJointEnergy | 1.510 | 1.09-2.1 | 0.01 |
| 245 | wavelet-HLHglcmMaximumProbability | 1.440 | 1.02-2.04 | 0.04 |
| 246 | wavelet-HLHgldmDependenceNonUniformity | 1.990 | 1.5-2.64 | <0.001 |
| 247 | wavelet-HLHgldmDependenceVariance | 1.470 | 1.07-2.01 | 0.02 |
| 248 | wavelet-HLHgldmGrayLevelNonUniformity | 2.310 | 1.7-3.12 | <0.001 |
| 249 | wavelet-HLHgldmLargeDependenceEmphasis | 1.560 | 1.12-2.17 | 0.008 |
| 250 | wavelet-HLHgldmLargeDependenceHighGrayLevelEmphasis | 1.770 | 1.2-2.6 | 0.004 |
| 251 | wavelet-HLHglrlmGrayLevelNonUniformity | 2.360 | 1.72-3.22 | <0.001 |
| 252 | wavelet-HLHglrlmLongRunEmphasis | 1.560 | 1.12-2.16 | 0.008 |
| 253 | wavelet-HLHglrlmRunLengthNonUniformity | 2.160 | 1.61-2.9 | <0.001 |
| 254 | wavelet-HLHglrlmRunLengthNonUniformityNormalized | 0.640 | 0.45-0.91 | 0.01 |
| 255 | wavelet-HLHglrlmRunPercentage | 0.640 | 0.45-0.9 | 0.01 |
| 256 | wavelet-HLHglrlmRunVariance | 1.550 | 1.12-2.14 | 0.008 |
| 257 | wavelet-HLHglrlmShortRunEmphasis | 0.640 | 0.46-0.91 | 0.01 |
| 258 | wavelet-HLHglszmGrayLevelNonUniformity | 2.100 | 1.57-2.81 | <0.001 |
| 259 | wavelet-HLHglszmLargeAreaEmphasis | 2.010 | 1.46-2.77 | <0.001 |
| 260 | wavelet-HLHglszmLargeAreaHighGrayLevelEmphasis | 2.400 | 1.61-3.56 | <0.001 |
| 261 | wavelet-HLHglszmLargeAreaLowGrayLevelEmphasis | 1.440 | 1.06-1.94 | 0.02 |
| 262 | wavelet-HLHglszmSizeZoneNonUniformity | 2.050 | 1.53-2.74 | <0.001 |
| 263 | wavelet-HLHglszmZoneVariance | 2.010 | 1.46-2.77 | <0.001 |
| 264 | wavelet-HLHngtdmBusyness | 2.300 | 1.65-3.2 | <0.001 |
| 265 | wavelet-HLHngtdmContrast | 0.570 | 0.33-0.99 | 0.047 |
| 266 | wavelet-HLHngtdmStrength | 0.300 | 0.09-0.96 | 0.04 |
| 267 | wavelet-HHLfirstorderEnergy | 1.820 | 1.38-2.4 | <0.001 |
| 268 | wavelet-HHLfirstorderKurtosis | 1.930 | 1.21-3.06 | 0.006 |
| 269 | wavelet-HHLfirstorderMaximum | 1.590 | 1.07-2.35 | 0.02 |
| 270 | wavelet-HHLfirstorderMinimum | 0.560 | 0.38-0.85 | 0.006 |
| 271 | wavelet-HHLfirstorderRange | 1.710 | 1.14-2.56 | 0.009 |
| 272 | wavelet-HHLfirstorderTotalEnergy | 1.800 | 1.37-2.37 | <0.001 |
| 273 | wavelet-HHLglcmAutocorrelation | 1.810 | 1.24-2.62 | 0.002 |
| 274 | wavelet-HHLglcmIdn | 1.630 | 1.01-2.65 | 0.047 |
| 275 | wavelet-HHLglcmJointAverage | 1.780 | 1.18-2.67 | 0.006 |
| 276 | wavelet-HHLglcmSumAverage | 1.780 | 1.18-2.67 | 0.006 |
| 277 | wavelet-HHLgldmDependenceNonUniformity | 2.010 | 1.51-2.67 | <0.001 |
| 278 | wavelet-HHLgldmGrayLevelNonUniformity | 1.980 | 1.51-2.58 | <0.001 |
| 279 | wavelet-HHLgldmHighGrayLevelEmphasis | 1.790 | 1.24-2.59 | 0.002 |
| 280 | wavelet-HHLgldmLargeDependenceHighGrayLevelEmphasis | 3.260 | 1.7-6.26 | <0.001 |

| | | | | |
|---|---|---|---|---|
| 281 | wavelet-HHLgldmSmallDependenceHighGrayLevelEmphasis | 1.390 | 1.01-1.92 | 0.04 |
| 282 | wavelet-HHLglrlmGrayLevelNonUniformity | 2.030 | 1.54-2.67 | <0.001 |
| 283 | wavelet-HHLglrlmHighGrayLevelRunEmphasis | 1.790 | 1.24-2.59 | 0.002 |
| 284 | wavelet-HHLglrlmLongRunHighGrayLevelEmphasis | 2.180 | 1.46-3.25 | <0.001 |
| 285 | wavelet-HHLglrlmRunLengthNonUniformity | 2.190 | 1.63-2.96 | <0.001 |
| 286 | wavelet-HHLglrlmShortRunHighGrayLevelEmphasis | 1.730 | 1.2-2.5 | 0.003 |
| 287 | wavelet-HHLglszmGrayLevelNonUniformity | 2.120 | 1.58-2.84 | <0.001 |
| 288 | wavelet-HHLglszmHighGrayLevelZoneEmphasis | 1.790 | 1.23-2.6 | 0.002 |
| 289 | wavelet-HHLglszmLargeAreaEmphasis | 1.570 | 1.19-2.07 | 0.001 |
| 290 | wavelet-HHLglszmLargeAreaHighGrayLevelEmphasis | 2.390 | 1.49-3.83 | <0.001 |
| 291 | wavelet-HHLglszmSizeZoneNonUniformity | 1.980 | 1.49-2.63 | <0.001 |
| 292 | wavelet-HHLglszmSmallAreaHighGrayLevelEmphasis | 1.760 | 1.22-2.54 | 0.003 |
| 293 | wavelet-HHLglszmZoneVariance | 1.570 | 1.2-2.07 | 0.001 |
| 294 | wavelet-HHLngtdmBusyness | 1.470 | 1.13-1.92 | 0.005 |
| 295 | wavelet-HHLngtdmComplexity | 1.390 | 1.03-1.87 | 0.03 |
| 296 | wavelet-HHLngtdmStrength | 0.420 | 0.19-0.93 | 0.03 |
| 297 | wavelet-HHHfirstorderEnergy | 1.810 | 1.37-2.4 | <0.001 |
| 298 | wavelet-HHHfirstorderKurtosis | 1.760 | 1.11-2.81 | 0.02 |
| 299 | wavelet-HHHfirstorderMaximum | 1.590 | 1.11-2.27 | 0.01 |
| 300 | wavelet-HHHfirstorderMinimum | 0.590 | 0.41-0.84 | 0.004 |
| 301 | wavelet-HHHfirstorderRange | 1.690 | 1.16-2.44 | 0.006 |
| 302 | wavelet-HHHfirstorderTotalEnergy | 1.800 | 1.36-2.38 | <0.001 |
| 303 | wavelet-HHHglcmAutocorrelation | 1.560 | 1.18-2.08 | 0.002 |
| 304 | wavelet-HHHglcmIdn | 1.610 | 1-2.58 | 0.048 |
| 305 | wavelet-HHHglcmJointAverage | 1.690 | 1.18-2.42 | 0.004 |
| 306 | wavelet-HHHglcmSumAverage | 1.690 | 1.18-2.42 | 0.004 |
| 307 | wavelet-HHHgldmDependenceNonUniformity | 2.020 | 1.52-2.69 | <0.001 |
| 308 | wavelet-HHHgldmGrayLevelNonUniformity | 1.920 | 1.48-2.5 | <0.001 |
| 309 | wavelet-HHHgldmHighGrayLevelEmphasis | 1.570 | 1.18-2.09 | 0.002 |
| 310 | wavelet-HHHgldmLargeDependenceHighGrayLevelEmphasis | 1.690 | 1.27-2.24 | <0.001 |
| 311 | wavelet-HHHgldmSmallDependenceHighGrayLevelEmphasis | 1.460 | 1.07-1.99 | 0.02 |
| 312 | wavelet-HHHglrlmGrayLevelNonUniformity | 2.040 | 1.55-2.69 | <0.001 |
| 313 | wavelet-HHHglrlmHighGrayLevelRunEmphasis | 1.570 | 1.18-2.09 | 0.002 |
| 314 | wavelet-HHHglrlmLongRunHighGrayLevelEmphasis | 1.600 | 1.22-2.1 | 0.001 |
| 315 | wavelet-HHHglrlmRunLengthNonUniformity | 2.140 | 1.6-2.87 | <0.001 |
| 316 | wavelet-HHHglrlmShortRunHighGrayLevelEmphasis | 1.560 | 1.17-2.09 | 0.003 |
| 317 | wavelet-HHHglszmGrayLevelNonUniformity | 2.150 | 1.6-2.88 | <0.001 |
| 318 | wavelet-HHHglszmGrayLevelVariance | 1.330 | 1-1.78 | 0.049 |
| 319 | wavelet-HHHglszmHighGrayLevelZoneEmphasis | 1.570 | 1.18-2.09 | 0.002 |
| 320 | wavelet-HHHglszmLargeAreaEmphasis | 1.550 | 1.18-2.02 | 0.002 |
| 321 | wavelet-HHHglszmLargeAreaHighGrayLevelEmphasis | 1.730 | 1.32-2.26 | <0.001 |
| 322 | wavelet-HHHglszmSizeZoneNonUniformity | 1.980 | 1.48-2.64 | <0.001 |
| 323 | wavelet-HHHglszmSmallAreaHighGrayLevelEmphasis | 1.570 | 1.18-2.09 | 0.002 |
| 324 | wavelet-HHHglszmZoneVariance | 1.550 | 1.18-2.02 | 0.002 |
| 325 | wavelet-HHHngtdmComplexity | 1.340 | 1.04-1.72 | 0.02 |
| 326 | wavelet-LLLfirstorder10Percentile | 2.730 | 1.79-4.15 | <0.001 |
| 327 | wavelet-LLLfirstorder90Percentile | 2.640 | 1.05-6.68 | 0.04 |

| | | | | |
|---|---|---|---|---|
| 328 | wavelet-LLLfirstorderEnergy | 1.690 | 1.11-2.58 | 0.02 |
| 329 | wavelet-LLLfirstorderEntropy | 0.360 | 0.23-0.57 | <0.001 |
| 330 | wavelet-LLLfirstorderInterquartileRange | 0.550 | 0.35-0.85 | 0.007 |
| 331 | wavelet-LLLfirstorderKurtosis | 1.390 | 1.08-1.79 | 0.01 |
| 332 | wavelet-LLLfirstorderMeanAbsoluteDeviation | 0.540 | 0.34-0.86 | 0.009 |
| 333 | wavelet-LLLfirstorderMean | 2.950 | 1.63-5.35 | <0.001 |
| 334 | wavelet-LLLfirstorderMedian | 2.510 | 1.41-4.48 | 0.002 |
| 335 | wavelet-LLLfirstorderRobustMeanAbsoluteDeviation | 0.520 | 0.33-0.83 | 0.005 |
| 336 | wavelet-LLLfirstorderRootMeanSquared | 0.310 | 0.18-0.55 | <0.001 |
| 337 | wavelet-LLLfirstorderSkewness | 0.300 | 0.18-0.51 | <0.001 |
| 338 | wavelet-LLLfirstorderTotalEnergy | 1.640 | 1.11-2.44 | 0.01 |
| 339 | wavelet-LLLfirstorderUniformity | 3.040 | 1.96-4.7 | <0.001 |
| 340 | wavelet-LLLglcmAutocorrelation | 2.070 | 1.34-3.2 | 0.001 |
| 341 | wavelet-LLLglcmContrast | 0.470 | 0.28-0.81 | 0.007 |
| 342 | wavelet-LLLglcmDifferenceAverage | 0.400 | 0.24-0.66 | <0.001 |
| 343 | wavelet-LLLglcmDifferenceEntropy | 0.370 | 0.23-0.59 | <0.001 |
| 344 | wavelet-LLLglcmId | 2.610 | 1.65-4.13 | <0.001 |
| 345 | wavelet-LLLglcmIdm | 2.580 | 1.65-4.05 | <0.001 |
| 346 | wavelet-LLLglcmIdmn | 2.030 | 1.29-3.19 | 0.002 |
| 347 | wavelet-LLLglcmIdn | 2.120 | 1.38-3.26 | 0.001 |
| 348 | wavelet-LLLglcmImc1 | 2.360 | 1.16-4.79 | 0.02 |
| 349 | wavelet-LLLglcmImc2 | 0.470 | 0.34-0.66 | <0.001 |
| 350 | wavelet-LLLglcmInverseVariance | 2.540 | 1.62-3.99 | <0.001 |
| 351 | wavelet-LLLglcmJointAverage | 2.320 | 1.42-3.79 | 0.001 |
| 352 | wavelet-LLLglcmJointEnergy | 2.920 | 1.86-4.58 | <0.001 |
| 353 | wavelet-LLLglcmJointEntropy | 0.620 | 0.42-0.92 | 0.02 |
| 354 | wavelet-LLLglcmMaximumProbability | 2.230 | 1.52-3.27 | <0.001 |
| 355 | wavelet-LLLglcmSumAverage | 2.320 | 1.42-3.79 | 0.001 |
| 356 | wavelet-LLLglcmSumEntropy | 0.380 | 0.24-0.61 | <0.001 |
| 357 | wavelet-LLLglcmSumSquares | 0.560 | 0.33-0.97 | 0.04 |
| 358 | wavelet-LLLgldmDependenceNonUniformity | 2.190 | 1.6-2.99 | <0.001 |
| 359 | wavelet-LLLgldmDependenceNonUniformityNormalized | 0.420 | 0.25-0.68 | 0.001 |
| 360 | wavelet-LLLgldmDependenceVariance | 1.800 | 1.29-2.51 | 0.001 |
| 361 | wavelet-LLLgldmGrayLevelNonUniformity | 2.610 | 1.86-3.66 | <0.001 |
| 362 | wavelet-LLLgldmHighGrayLevelEmphasis | 2.100 | 1.37-3.23 | 0.001 |
| 363 | wavelet-LLLgldmLargeDependenceEmphasis | 2.550 | 1.69-3.84 | <0.001 |
| 364 | wavelet-LLLgldmLargeDependenceHighGrayLevelEmphasis | 2.540 | 1.69-3.83 | <0.001 |
| 365 | wavelet-LLLgldmLargeDependenceLowGrayLevelEmphasis | 0.350 | 0.15-0.84 | 0.02 |
| 366 | wavelet-LLLgldmLowGrayLevelEmphasis | 0.100 | 0.02-0.51 | 0.006 |
| 367 | wavelet-LLLgldmSmallDependenceEmphasis | 0.380 | 0.24-0.61 | <0.001 |
| 368 | wavelet-LLLgldmSmallDependenceLowGrayLevelEmphasis | 0.090 | 0.01-0.65 | 0.02 |
| 369 | wavelet-LLLglrlmGrayLevelNonUniformity | 2.430 | 1.77-3.34 | <0.001 |
| 370 | wavelet-LLLglrlmGrayLevelNonUniformityNormalized | 2.990 | 1.94-4.6 | <0.001 |
| 371 | wavelet-LLLglrlmHighGrayLevelRunEmphasis | 2.100 | 1.37-3.22 | 0.001 |
| 372 | wavelet-LLLglrlmLongRunEmphasis | 2.770 | 1.79-4.28 | <0.001 |
| 373 | wavelet-LLLglrlmLongRunHighGrayLevelEmphasis | 2.360 | 1.52-3.65 | <0.001 |
| 374 | wavelet-LLLglrlmLongRunLowGrayLevelEmphasis | 0.110 | 0.02-0.53 | 0.006 |

| | | | | |
|---|---|---|---|---|
| 375 | wavelet-LLLglrlmLowGrayLevelRunEmphasis | 0.100 | 0.02-0.52 | 0.006 |
| 376 | wavelet-LLLglrlmRunEntropy | 0.400 | 0.26-0.63 | <0.001 |
| 377 | wavelet-LLLglrlmRunLengthNonUniformity | 2.290 | 1.68-3.14 | <0.001 |
| 378 | wavelet-LLLglrlmRunLengthNonUniformityNormalized | 0.370 | 0.23-0.58 | <0.001 |
| 379 | wavelet-LLLglrlmRunPercentage | 0.370 | 0.24-0.57 | <0.001 |
| 380 | wavelet-LLLglrlmRunVariance | 2.660 | 1.75-4.06 | <0.001 |
| 381 | wavelet-LLLglrlmShortRunEmphasis | 0.360 | 0.23-0.56 | <0.001 |
| 382 | wavelet-LLLglrlmShortRunHighGrayLevelEmphasis | 2.040 | 1.33-3.12 | 0.001 |
| 383 | wavelet-LLLglrlmShortRunLowGrayLevelEmphasis | 0.100 | 0.02-0.51 | 0.006 |
| 384 | wavelet-LLLglszmGrayLevelNonUniformity | 2.020 | 1.53-2.67 | <0.001 |
| 385 | wavelet-LLLglszmGrayLevelNonUniformityNormalized | 1.830 | 1.27-2.64 | 0.001 |
| 386 | wavelet-LLLglszmHighGrayLevelZoneEmphasis | 2.030 | 1.35-3.06 | 0.001 |
| 387 | wavelet-LLLglszmLargeAreaEmphasis | 3.260 | 1.95-5.43 | <0.001 |
| 388 | wavelet-LLLglszmLargeAreaHighGrayLevelEmphasis | 2.970 | 1.9-4.65 | <0.001 |
| 389 | wavelet-LLLglszmLargeAreaLowGrayLevelEmphasis | 3.540 | 1.99-6.31 | <0.001 |
| 390 | wavelet-LLLglszmLowGrayLevelZoneEmphasis | 0.130 | 0.03-0.66 | 0.01 |
| 391 | wavelet-LLLglszmSizeZoneNonUniformity | 2.370 | 1.68-3.34 | <0.001 |
| 392 | wavelet-LLLglszmSizeZoneNonUniformityNormalized | 0.530 | 0.35-0.8 | 0.002 |
| 393 | wavelet-LLLglszmSmallAreaEmphasis | 0.520 | 0.35-0.77 | 0.001 |
| 394 | wavelet-LLLglszmSmallAreaHighGrayLevelEmphasis | 1.880 | 1.25-2.84 | 0.003 |
| 395 | wavelet-LLLglszmSmallAreaLowGrayLevelEmphasis | 0.160 | 0.03-0.84 | 0.03 |
| 396 | wavelet-LLLglszmZonePercentage | 0.390 | 0.24-0.61 | <0.001 |
| 397 | wavelet-LLLglszmZoneVariance | 3.260 | 1.95-5.43 | <0.001 |
| 398 | wavelet-LLLngtdmBusyness | 2.670 | 1.83-3.91 | <0.001 |
| 399 | wavelet-LLLngtdmContrast | 0.480 | 0.29-0.79 | 0.004 |
| 400 | wavelet-LLLngtdmStrength | 0.420 | 0.19-0.92 | 0.03 |

**Supplementary Table S6 Radiomic features selected by LASSO-Cox regression**

| | **Feature** | **Coefficient** |
|---|---|---|
| 1 | wavelet-LLHngtdmBusyness | 0.336 |
| 2 | wavelet-LLLfirstorderSkewness | -0.082 |
| 3 | wavelet-LLLfirstorderUniformity | 0.122 |
| 4 | wavelet-LLLglcmMaximumProbability | 0.023 |
| 5 | wavelet-LLLgldmGrayLevelNonUniformity | 0.247 |
| | intercept | 0.000 |

**Supplementary Table S7 Univariate Cox regression analysis of clinicopathological features**

| **Feature** | **Hazard ratio** | **95% CI** | ***p*-value** |
|---|---|---|---|
| HLA-I | 1.000 | 0.99-1.01 | 0.69 |
| PD-L1 | 1.000 | 0.99-1.01 | 0.88 |
| CD8 | 0.950 | 0.9-1 | 0.052 |
| Sex | 0.430 | 0.19-1 | **0.049** |
| Age | 1.030 | 0.99-1.08 | 0.16 |
| Smoking History | 1.940 | 0.9-4.19 | 0.09 |
| T Stage | 2.960 | 1.36-6.4 | **0.006** |
| N Stage | 5.310 | 2.34-12.02 | **0.000** |
| Pathological Stage | 4.130 | 1.86-9.14 | **0.000** |

Bolded values indicate a statistically significant result.

**Supplementary Table S8 Multivariate Cox regression analyses of clinicopathological features and RAD-risk score**

| **Feature** | **Hazard ratio** | **95% CI** | ***p*-value** |
|---|---|---|---|
| RAD-risk score | 2.31 | 1.61-3.32 | <0.001 |
| N stage | 2.32 | 0.88-6.10 | 0.09 |
