# Supplementary material for: Radiomic features and tumor immune microenvironment associated with anaplastic lymphoma kinase-rearranged lung adenocarcinoma and their prognostic value
Source: Front Genet. 2025 May 1;16:1581937. doi: 10.3389/fgene.2025.1581937 (PMC12078255; doi:10.3389/fgene.2025.1581937)
Supplement: Supplementary file 3 [file DataSheet1.pdf]

Supplement 1

1. **Original.GLCM.Autocorrelation:** A Gray Level Co-Occurrence Matrix（**GLCM**）based texture feature **Autocorrelation**.

GLCM is defined as $P(i,j;\delta,\alpha)$, a matrix with size $N_g \times N_g$ describing the second-order joint probability function of an image, where the (i,j) th element represents the number of times the combination of intensity levels i and j occur in two pixels in the image, that are separated by a distance of δ pixels in direction α, and N_g is the number of discrete gray level intensities.

Autocorrelation is calculated with the following formula:

$$autocorrelation = \sum_{i=1}^{N_g}\sum_{j=1}^{N_g} ijP(i,j)$$

2. **Wavelet_LLH.GLSZM.SmallAreaEmphasis:** A Wavelet-based feature transformed from Gray Level Size Zone Matrix (**GLSZM**) feature **Small Area Emphasis (SAE).**

Wavelet transform effectively decouples textural information by decomposing the original image, in a similar manner as Fourier analysis, in low- and high- frequencies. In this study, a discrete, one-level and undecimated three-dimensional wavelet transform was applied to each CT image, which decomposes the original image X into eight decompositions. Consider L and H to be a low-pass (i.e. a scaling) and, respectively, a high-pass (i.e. a wavelet) function, and the wavelet decompositions of X to be labeled as X_{LLL}, X_{LLH}, X_{LHL}, X_{LHH}, X_{HLL}, X_{HLH}, X_{HHL} and X_{HHH}. For example, X_{LLH} is then interpreted as the high-pass sub-band, resulting from directional filtering of X with a low-pass filter along x-direction, a low-pass filter along y-direction and a high-pass filter along z-direction and is constructed as:

$$X_{LLH}(i,j,k) = \sum_{p=1}^{N_L}\sum_{q=1}^{N_L}\sum_{r=1}^{N_H} L(p)L(q)H(r)X(i+p,j+q,k+r)$$

Where N_L is the length of filter L and N_H is the length of filter H. The other decompositions are constructed in a similar manner, applying their respective ordering of low or high-pass filtering in x, y and z-direction.

GLSZM quantifies size zone matrices in an image. Instead of looking in several directions as GLCM, it looks at flat zone size for the whole image. A flat zone is a group of connecting pixels with the same gray level. In a gray level size matrix $p(i,j)$, the (i,j)th element describes the frequency of matrices of size j with gray level i, and N_g is the number of discrete gray level intensities.

SAE is calculated with the following formula:

$$SAE = \frac{\sum_{i=1}^{N_g}\sum_{j=1}^{N_r}\left[\frac{p(i,j)}{j^2}\right]}{\sum_{i=1}^{N_g}\sum_{j=1}^{N_r}p(i,j)}$$

3. **Wavelet_HLL.GLCM.Correlation:** A Wavelet-based feature transformed from **GLCM** feature **Correlation.**

Correlation is calculated with the following formula:

$$correlation = \frac{\sum_{i=1}^{N_g}\sum_{j=1}^{N_g} ijP(i,j) - \mu_i(i)\mu_j(j)}{\sigma_x(i)\sigma_y(j)}$$

4. **Wavelet_LLL.Firstorder.Skewness:** A Wavelet-based feature transformed from **First-order** feature **Skewness**.

First-order features describe the distribution of voxel intensities within the CT image through commonly used and basic metrics. Let X denote the three-dimensional image matrix with N voxels and P the first order histogram divided by N_l discrete intensity levels.

Skewness is calculated with the following formula:

$$skewness = \frac{\frac{1}{Np}\sum_{i=1}^{Np}(X(i)-\bar{X})^3}{\left(\sqrt{\frac{1}{Np}\sum_{i=1}^{Np}(X(i)-\bar{X})^2}\right)^3}$$

5. **Wavelet_LLH.NGTDM.Busyness:** A Wavelet-based feature transformed from neighboring gray-tone difference matrix **(NGTDM)** feature **Busyness.**

NGTDM quantifies the difference between a gray value and the average gray value of its neighbors within distance δ. The sum of absolute differences for gray level i is stored in the matrix.

Busyness is calculated with the following formula:

$$Busyness = \frac{\sum_{i=1}^{N_g} pi\, si}{\sum_{i=1}^{N_g} \sum_{j=1}^{N_g} |ipi - jpj|}$$

6. **Wavelet_LLL.GLCM.MaximumProbability:** A Wavelet-based feature transformed from GLCM feature **Maximum Probability.**

$$maximum\ probability = \max\{P(i,j)\}$$

Reference:

Coroller, T.P., Grossmann, P., Hou, Y., Rios Velazquez, E., Leijenaar, R.T., Hermann, G., et al. (2015). CT-based radiomic signature predicts distant metastasis in lung adenocarcinoma. *Radiother Oncol.* 114**,** 345-350. doi: 10.1016/j.radonc.2015.02.015

For the mathematical formulas of the remaining studied radiomics features, see https://pyradiomics.readthedocs.io/en/latest/features.html.
